# Increasing autophagy and blocking Nrf2 suppress laminopathy‐induced age‐dependent cardiac dysfunction and shortened lifespan

**DOI:** 10.1111/acel.12747

**Published:** 2018-03-25

**Authors:** Shruti Bhide, Adriana S. Trujillo, Maureen T. O'Connor, Grant H. Young, Diane E. Cryderman, Sahaana Chandran, Mastaneh Nikravesh, Lori L. Wallrath, Girish C. Melkani

**Affiliations:** ^1^ Department of Biology, Molecular Biology and Heart Institutes San Diego State University San Diego CA USA; ^2^ Department of Biochemistry Carver College of Medicine University of Iowa Iowa City IA USA

**Keywords:** autophagy, cardiac aging, Drosophila aging model, lamins, Nrf2/Keap1 pathway, protein aggregation

## Abstract

Mutations in the human *LMNA* gene cause a collection of diseases known as laminopathies. These include myocardial diseases that exhibit age‐dependent penetrance of dysrhythmias and heart failure. The *LMNA* gene encodes A‐type lamins, intermediate filaments that support nuclear structure and organize the genome. Mechanisms by which mutant lamins cause age‐dependent heart defects are not well understood. To address this issue, we modeled human disease‐causing mutations in the *Drosophila melanogaster Lamin C* gene and expressed mutant *Lamin C* exclusively in the heart. This resulted in progressive cardiac dysfunction, loss of adipose tissue homeostasis, and a shortened adult lifespan. Within cardiac cells, mutant *Lamin C* aggregated in the cytoplasm, the CncC(Nrf2)/Keap1 redox sensing pathway was activated, mitochondria exhibited abnormal morphology, and the autophagy cargo receptor Ref2(P)/p62 was upregulated. Genetic analyses demonstrated that simultaneous over‐expression of the autophagy kinase *Atg1* gene and an RNAi against *CncC* eliminated the cytoplasmic protein aggregates, restored cardiac function, and lengthened lifespan. These data suggest that simultaneously increasing rates of autophagy and blocking the Nrf2/Keap1 pathway are a potential therapeutic strategy for cardiac laminopathies.

## 1 INTRODUCTION

Many characteristics of normal aging appear to be accelerated in individuals with dominant mutations in the *LMNA* gene encoding A‐type lamins (Ahmed, Ikram, Bibi & Mir, 2017; Apte, Stick & Radmacher, 2017; Cenni et al., 2017; Ikeda et al., 2016; Scaffidi & Misteli, 2006). These include cardiac diseases with a broad range of arrhythmic disturbances, left ventricle dysfunction, and heart failure that show increasing penetrance with age (Captur et al., 2017; Liang, Grogan & Ackerman, 2016). To gain insights into how to delay the onset and/or prevent these cardiac defects during aging, a greater understanding of the molecular basis of the pathology is needed.

The human *LMNA* gene encodes the developmentally regulated and nearly ubiquitously expressed A‐type lamins, Lamin A and C, which are produced by alternate splicing (Burke & Stewart, 2013). Lamins are intermediate filaments that line the inner nuclear membrane, providing structural support for the nucleus and organizing the genomic DNA (Ahmed et al., 2017; Azibani, Muchir, Vignier, Bonne & Bertrand, 2014; Guenantin et al., 2014; Wang, Zabell, Koh & Tang, 2017; Worman, 2012). Over 400 mutations have been identified in the *LMNA* gene (Dittmer & Misteli, 2011; Gruenbaum & Foisner, 2015). In addition to cardiac diseases, these mutations cause skeletal muscular dystrophy with age‐dependent penetrance and early‐onset aging syndromes; collectively, these diseases are referred to as laminopathies (Politano et al., 2013).

Genetically tractable model organisms have been used to understand the functions of lamins. Mice lacking A‐type lamins have severe cardiac and skeletal muscle defects, in addition to a shortened lifespan (Ramos et al., 2012; Zhang, Kieckhaefer & Cao, 2013). Furthermore, studies on the mouse models demonstrated that the cardiac defects and shortened lifespan can be partially reversed by treatment with rapamycin and temsirolimus (a derivative of rapamycin) (Choi et al., 2012; Ramos et al., 2012). Studies in Drosophila demonstrated that mutant lamins, modeled after those that cause human disease, lead to cytoplasmic aggregation of nuclear envelope proteins and loss of redox homeostasis in larval body wall muscles (Dialynas et al., 2012, 2015). Consistent with these findings, human muscle biopsy tissues showed both cytoplasmic aggregation of nuclear envelope (NE) proteins and activation of the Nrf2/Keap‐1 signaling pathway. Thus, these models have phenotypes similar to the human disease condition.

Here, we developed a Drosophila model of cardiac laminopathies. Mutations in the human *LMNA* that cause dilated cardiomyopathy with conduction defects are often point mutations resulting in amino acid substitutions in residues conserved among species. We modeled these mutations in the Drosophila *Lamin C* gene (hereafter referred as *LamC*) and assayed for effects on the fruit fly heart. Mechanisms of cardiac development and function are shared between Drosophila and humans (Diop & Bodmer, 2015; Melkani et al., 2013; Zhu et al., 2017). Furthermore, Drosophila has successfully been used to identify the genetic basis of cardiac deterioration that arises due to aging and metabolic dysregulation (Diop & Bodmer, 2015; Gill, Le, Melkani & Panda, 2015; Melkani et al., 2013).

The cardiolaminopathy Drosophila models exhibited age‐dependent decline in cardiac function that resulted in a shortened adult lifespan. Defects were observed in the nucleus, cytoplasm, and mitochondria of cardiomyocytes. In addition, adults showed an age‐dependent increase in triglycerides. Many of these abnormal features are common in human cardiac laminopathies (Captur et al., 2017). The Drosophila models allowed for genetic tests of suppression and identified new potential therapeutic targets for individuals with cardiolaminopathy and other types of laminopathies.

## 2 RESULTS

### 2.1 Mutant LamC caused age‐dependent cardiac defects and a shortened adult lifespan

**Figure 1:**
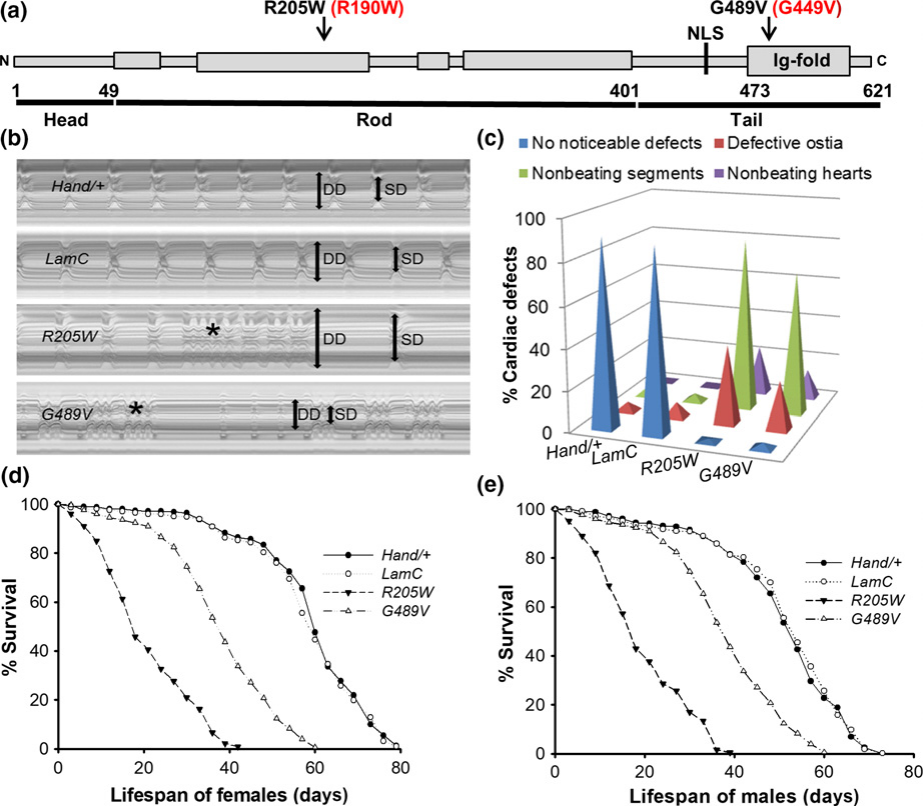
Drosophila Lamin C domain structure and effects of mutant LamC on cardiac function and lifespan. (a) Lamins contain a conserved structure with an N‐terminal head domain, coiled‐coil rod domain, and tail domain possessing an Ig‐fold. Amino acid substitutions in the rod and Ig‐fold domains used in this study are indicated. Numbering for Drosophila Lamin C is in black; the corresponding human disease‐causing amino acid substitution is in red. (b) M‐mode recordings (5‐s time periods) of dissected hearts from 3‐week‐old female *Hand‐Gal4/+*, wild‐type and mutant *Lamin C (LamC)* (*R205W* an*d G489V)*. M‐mode analysis revealed that *R205W‐*expressing hearts showed significant dilation and arrhythmias, whereas G489V‐expressing hearts showed restricted morphology and arrhythmias. Double‐headed arrows in the M‐mode traces indicate diastolic diameter (DD) and systolic diameter (SD) between the walls of the heart. (c) Summary of the qualitative cardiac defects from 3‐week‐old male and female adult controls (*Hand/+* and wild‐type *LamC*) and mutants (*R205W* and *G489V*) showing the percent of flies exhibiting defective ostia, one or more noncontractile regions (conductive defect), and nonbeating hearts. (d and e) Cardiac‐specific expression of mutant *LamC* (*R205W* and *G489V*) resulted in a reduction in lifespan compared to controls expressing *Hand*/+ and wild‐type *LamC* controls (*p* < .001). Graphs indicating the percent survival for female adults (*n * =  150 for each group) versus age days posteclosion

To determine the mechanistic basis of cardiolaminopathies, we expressed wild‐type and mutant Drosophila *LamC transgenes* (referred to as *R205W* and *G489V* hereafter) in the heart (Figure 1a). These amino acid substitutions are analogous to human Lamin A/C R190W and G449V, respectively. Mutations in *LMNA* that give rise to Lamin A/C R190W are associated with progressive cardiac defect (including conduction defects) and reduced cardiac performance (Arbustini et al., 2002; Heller et al., 2017; Hermida‐Prieto et al., 2004). Mutations in *LMNA* that give rise to Lamin A/C G449V cause congenital muscular dystrophy, which is characterized by skeletal muscle defects in childhood and age‐dependent dilated cardiomyopathy (Dialynas et al., 2015). Cardiac‐specific expression of Drosophila *LamC* was obtained using the Gal4/UAS system with a *Hand‐Gal4* driver (Han & Olson, 2005; Melkani et al., 2013)**.** Expression of mutant *LamC*, but not wild‐type, caused adult cardiac defects in flies possessing an otherwise wild‐type genetic background (Figure 1b, c)**.** Thus, mutant lamins caused dominant defects in these models, similar to the human disease condition (Dittmer & Misteli, 2011; Gruenbaum & Foisner, 2015).

Defective cardiac function was measured and quantitated according to established protocols (Gill et al., 2015; Melkani et al., 2013). M‐mode analysis was performed in which a specified region of one‐pixel width along the image of the heart was selected from each movie frame and aligned horizontally, generating a montage that portrays the movement of the heart walls over time (Figure 1b). Hearts from 3‐week‐old female flies expressing *R205W* and *G489V* showed significant dilation and restriction, respectively. Consistent with our finding, mutations in human *LMNA* result in dominant dilated, hypertrophic, and idiopathic cardiomyopathy (Arbustini et al., 2002; Heller et al., 2017; Marian, 2017). Furthermore, expression of mutant *LamC* resulted in dysrhythmic beating patterns when compared to hearts from age‐matched controls expressing wild‐type LamC and the *Hand‐Gal4* driver alone (Figure 1b).

Expression of the mutant *LamC* resulted in defective ostia, noncontractile region(s) of the heart, and loss of heartbeat, an indicator of conduction defects (Figure 1c), which are observed in human *LMNA* patients (Arbustini et al., 2002; Brayson & Shanahan, 2017; Malhotra & Mason, 2009; Wolf et al., 2008). In addition to severe cardiac defects, heart‐specific expression of *R205W* and *G489V* had a drastic impact on the lifespan for both female and male adults compared to controls (Figure 1d, e). The half‐life of females expressing *R205W* and *G489V* was 21 and 39 days, respectively, compared to 58 days for females expressing wild‐type *LamC* (Figure 1d). Similarly, the half‐life of males expressing *R205* and *G489V* was 18 and 38 days, respectively, compared to 56 days for males expressing wild‐type *LamC* (Figure 1e). Western analysis showed that similar levels of wild‐type and mutant *LamC* were expressed by the *Hand*‐*Gal4* driver (Fig. S1a). Transgenic flies expressing wild‐type *LamC* had less than twofold higher level of LamC compared with nontransgenic controls. Importantly, the slightly elevated level of wild‐type LamC did not produce cardiac defects (Figure 1b, c). In contrast, the mutant versions of LamC expressed at levels similar to the exogenous wild‐type LamC produced obvious cardiac defects (Figure 1b, c). Thus, the cardiac defects were caused by mutant LamC and not increased total amounts of LamC. Furthermore, cardiac‐specific expression of mutant LamC did not result in noncardiac muscle defects as measured by adult flight (Fig. S1b). Therefore, the phenotypes were restricted to the muscle tissue in which the mutant *LamC* was expressed.

### 2.2 Mutant LamC caused progressive cardiac physiological dysfunction

**Figure 2:**
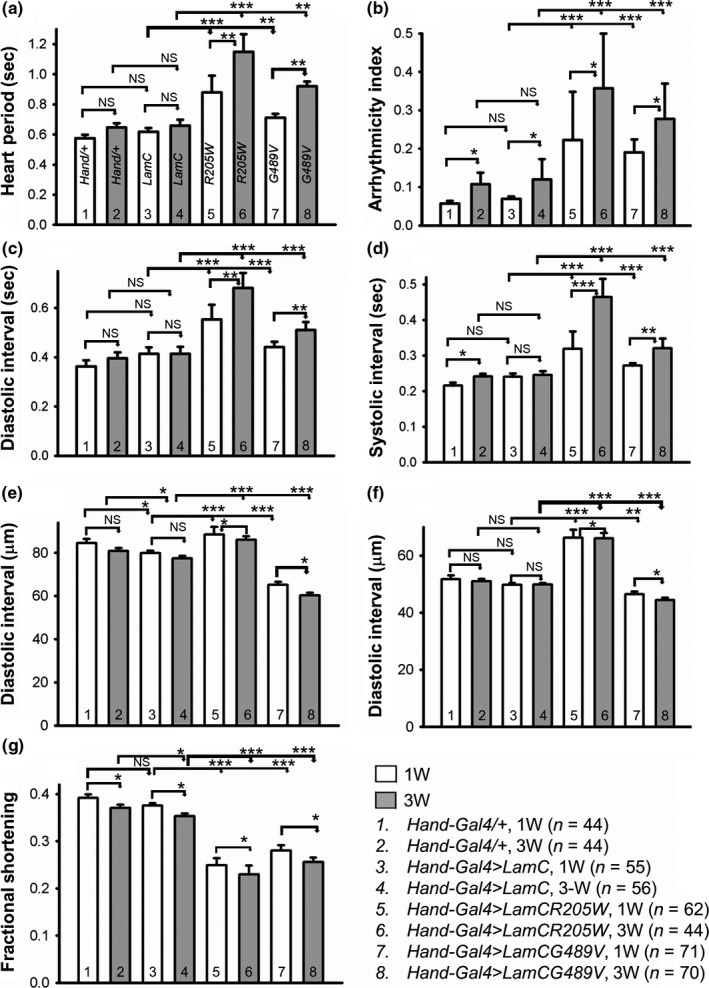
Mutant LamC caused progressive cardiac physiological dysfunction. One (1W) and three‐week (3W)‐old female adults (*n* = 44–71 per genotype) expressing *Hand‐Gal4/+*, wild‐type *LamC, R205W,* and *G489V* were assayed for the heart period (a), arrhythmia index (b), diastolic and systolic intervals (c and d), diastolic and systolic parameters (e and f), and fractional shortening (g). Minimal differences were observed between the *Hand/+* and wild‐type *LamC* for the cardiac parameters measured. In contrast, significant differences were observed for all parameters (a–g) between the mutant *LamC* (*R205W* and *G489V*) and age‐matched controls. Data are shown as average ± s.e.m.s; statistical significance was determined using one‐way ANOVA and Tukey's post hoc test. For all parameters, statistical significance is denoted *as follows*: *p <* *.05; **p <* *.01; ***p <* *.001; NS* *= not significant

Quantitative analysis of cardiac physiological parameters revealed progressive and severe cardiac defects upon expression of *R205W and G489V* (Figure 2). For example, the heart period (Figure 2a), arrhythmicity index (Figure 2b), diastolic interval (Figure 2c), and systolic interval (Figure 2d) of one‐ and 3‐week‐old female adults expressing *R205W* and *G489V* were significantly elevated compared to age‐matched controls. Furthermore, these parameters showed more severe deterioration in 3‐week‐old flies compared to one‐week‐old flies suggesting that the phenotypes were progressive (Figure 2a–d). Of note, only the systolic interval of hearts expressing the *Hand‐Gal4* driver alone and the arrhythmicity index of hearts expressing wild‐type *LamC* and the *Hand‐Gal4* driver alone showed subtle alterations in 3‐week‐old flies compared to their 1‐week‐old counterparts (Figure 2a, d). Due to the cardiac dilation caused by expression of *R205W* and the cardiac restriction caused by expression of *G489V*, both diastolic (Figure 2e) and systolic (Figure 2f) heart diameters were significantly enlarged and reduced at one and three weeks of age compared to age‐matched controls, respectively. The cardiac dilation and restriction reduced heart contractility (Figure 2e, f), which was further reflected by decreased fractional shortening (Figure 2g). Similar defects were observed in adult males (data not shown). Thus, these data demonstrate that heart‐specific expression of mutant *LamC* caused severe and progressive contractility‐related cardiac phenotypes.

### 2.3 Mutant LamC caused nuclear blebbing, cytoplasmic aggregation of LamC and Otefin (Emerin), and disruption of the myofibrils

**Figure 3:**
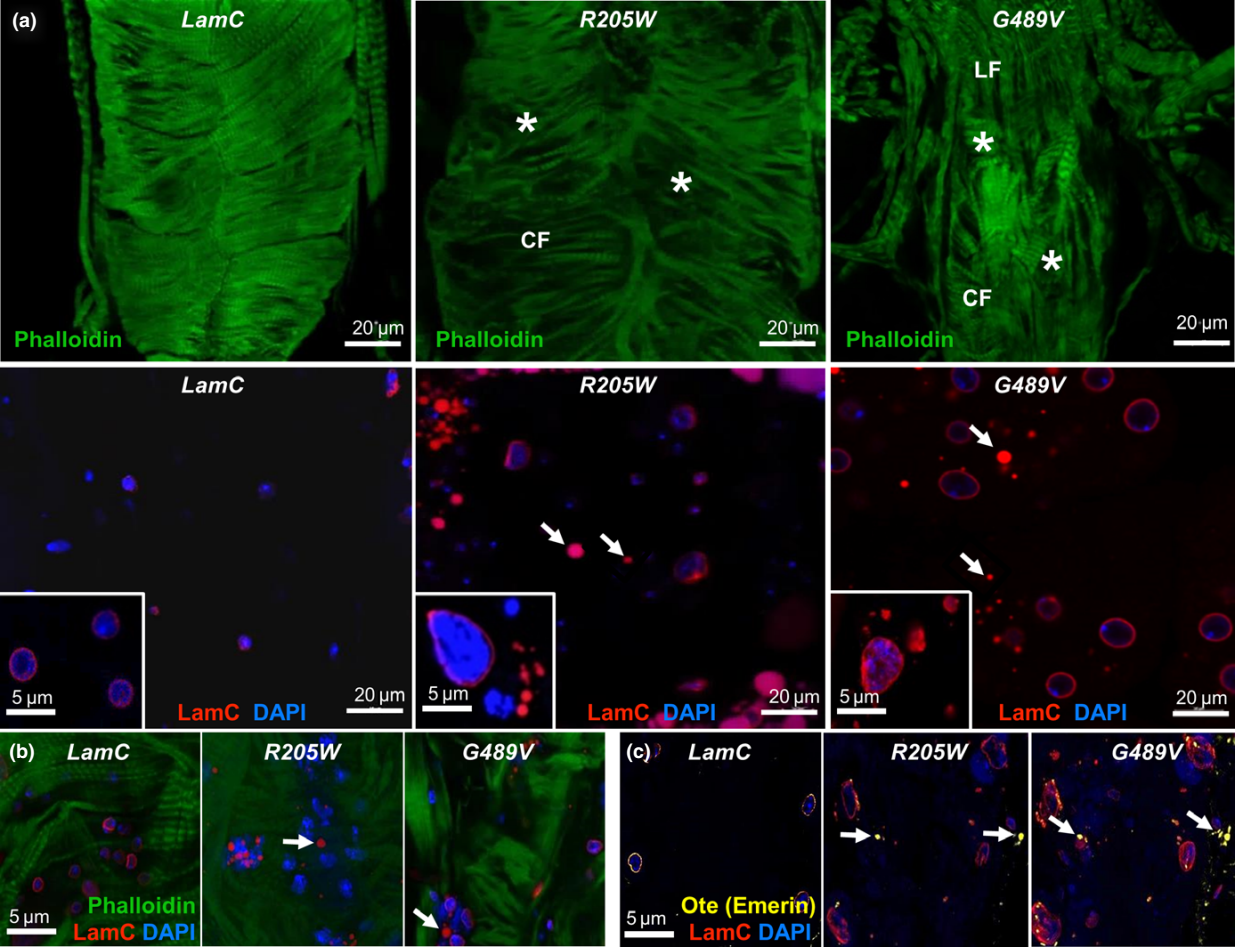
Mutant LamC caused myofibrillar disorganization, nuclear blebbing, and cytoplasmic aggregation of LamC and Otefin (orthologue of human Emerin). (a) Immunofluorescence of 3‐week‐old hearts stained with antibodies to LamC (red), phalloidin (F‐actin, green), and DAPI (DNA, blue). *R205W* and *G489V* caused disorganization of actin‐containing myofibrils that was not seen upon expression of wild‐type *LamC* (upper panels). CF and LF represent circumferential and longitudinal fibers, respectively. Asterisks represent myofibrillar disorganization (upper panels). Hearts expressing *R205W* and *G489V* that were stained with DAPI and anti‐LamC antibodies showed enlarged nuclei and cytoplasmic aggregation of LamC (arrows, lower panels). High magnification images are shown as insets. (b) Merged images of hearts expressing wild‐type *LamC*,* R205W,* and *G489V* stained with anti‐LamC antibodies (red) and DAPI (blue) showed cytoplasmic LamC aggregates (arrows) (c) Merged images of hearts expressing wild‐type *LamC, R205W,* and *G489V* stained with DAPI (DNA), anti‐LamC antibodies (red), and antibodies that recognize Otefin (yellow) showed cytoplasmic aggregates (arrows)

To examine the effects of mutant *LamC* expression at the cytological level, dissected hearts from three‐week‐old flies were fixed under relaxed conditions, incubated with antibodies specific for nuclear envelope proteins and/or fluorescent dyes to cytoskeletal proteins, and analyzed by confocal microscopy. Expression of wild‐type *LamC* showed normal nuclear morphology; LamC localized to the nuclear periphery and actin‐containing myofibrils were organized into a network (Figures 3a, b and S2a, b). In contrast, expression of the mutant *LamC* caused nuclear envelope blebbing, cytoplasmic aggregation of LamC, and disorganization of actin‐containing myofibrils (Figure 3a, b). The myofibrillar disorganization and LamC aggregation increased with age (Fig. S2a–c). Quantitation of LamC aggregates showed that the relative area occupied by the aggregates per total area surveyed was greater in hearts expressing the mutant *LamC* versus those expressing wild‐type *LamC* (Fig. S2c). Furthermore, the relative area occupied by aggregates increased with age (Fig. S2c). Immunostaining of hearts expressing wild‐type *LamC* with antibodies to Otefin (Ote) [a Drosophila orthologue of the human inner nuclear membrane LEM domain protein emerin (Barton, Lovander, Pinto & Geyer, 2016)] showed localization to the nuclear envelope as anticipated (Figure 3c). In contrast, hearts expressing mutant *LamC* showed greater amounts of cytoplasmic aggregation of Ote (Figures 3c and S2c). Thus, mutant LamC caused myofibril disorganization, nuclear blebbing, and cytoplasmic aggregation of nuclear envelope proteins.

**Figure 4:**
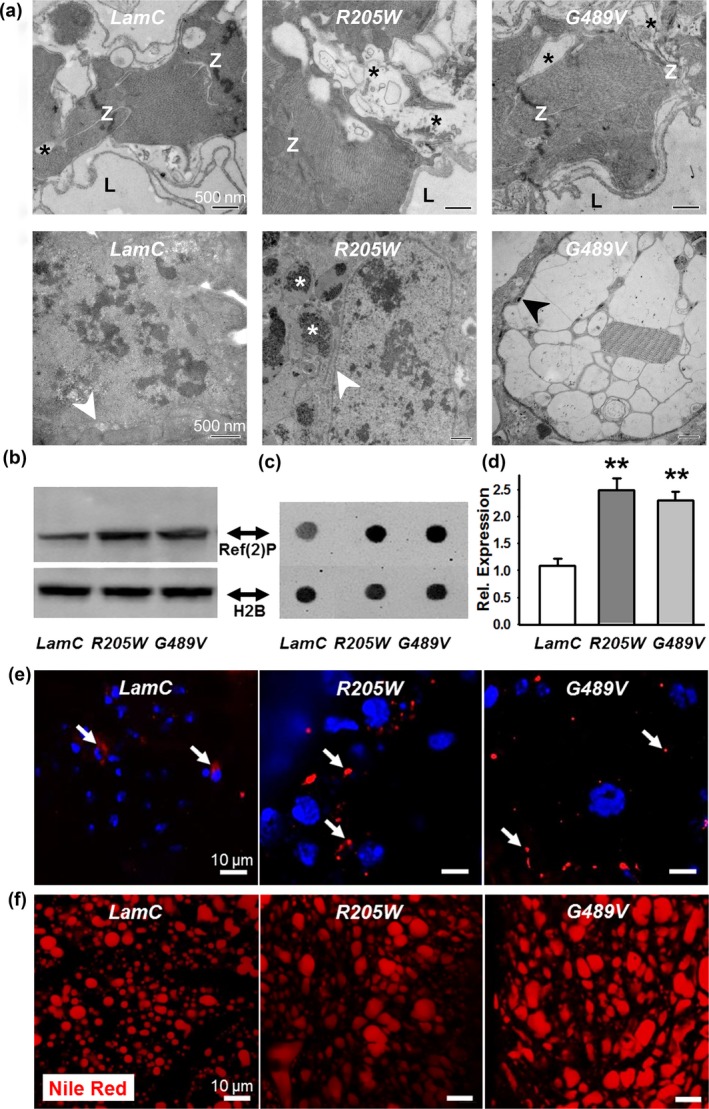
Cardiac‐specific expression of mutant LamC caused ultrastructural defects, upregulation of Ref(2)P, and altered adipose tissue homeostasis. (a) TEM micrographs of transverse sections through the heart of 3‐week‐old adults expressing wild‐type LamC showed contractile cardiomyocytes with characteristic Z‐disks (Z). Note that only minor myofibrillar degeneration was observed (black asterisk). In contrast, hearts expressing *R205W* and *G489V* showed substantial myofibrillar degeneration (black asterisks) and poorly organized Z‐disks (Z). The lumen (L) is a hemolymph‐containing compartment surrounded by contractile cardiomyocytes. In 3‐week‐old control hearts, intact membrane‐bound nuclei were detected. In contrast, hearts expressing *R205W* showed nuclear material outside of membrane‐bound nuclei (white asterisks). White arrows indicate the nuclear envelope. No intact nuclei were observed in 3‐week‐old hearts expressing *G489V;* however, membrane‐bound (black arrow) electron‐sparse structures were detected, which may constitute degenerated nuclei and/or vacuoles. Scale bars are 500 nm. (b) Western analysis of protein extracts from dissected hearts of 3‐week‐old female females expressing wild‐type and mutant LamC stained with antibodies that recognize Ref(2)P and histone H2B (loading control). (c) Representative antibody “dot blot” of protein extract from dissected hearts and stained with antibodies that recognize Ref(2)P and histone H2B. (d) Quantification of Ref(2)P staining, normalized to levels of histone H2B, in 3‐week‐old female flies hearts expressing *R205W* and *G489V* compared to same age flies expressing wild‐type *LamC* (**p < .01, *n* = 3 independent samples). (e) Cardiomyocytes of 3‐week‐old adults were stained with DAPI (blue) and antibodies to Ref(2)P (red). Hearts expressing mutant LamC showed Ref(2)P foci (arrows) that increased in number and size compared to those in flies expressing wild‐type *LamC*. (f) Adult fat bodies stained with Nile Red showed increased numbers and size of lipid droplets in expressing mutant *LamC* in cardiac tissue, relative to those expressing wild‐type *LamC* (nonautonomous effect)

To determine the effects of LamC aggregation in cardiac tissue at the ultrastructural level, we employed transmission electron microscopy (TEM) (Figure 4a). TEM micrographs of a transverse section through the dorsal vessel of 3‐week‐old adults were prepared according to published procedures (Melkani et al., 2013). Adults expressing wild‐type *LamC* showed cardiomyocytes with characteristic discontinuous Z‐disks (“Z”). In contrast, similar aged adults expressing mutant *LamC* possessed hearts with severe myofibrillar degeneration (black asterisks) and poorly organized Z‐disks (Figure 4a**,** upper panels). Control hearts contained intact membrane‐bound nuclei (Figure 4a, white arrowhead, lower left panel), while hearts expressing *R205W* contained nuclear material (white asterisks) outside of membrane‐bound nuclei (Figure 4a, white arrowhead, lower middle panel). No intact nuclei were observed in hearts of similarly aged adults expressing *G489V;* however, membrane‐bound (black arrowhead) electron‐sparse structures were detected, which might represent degenerated nuclei and/or vacuoles (Figure 4a, lower right panel). Thus, the ultrastructural data were consistent with the abnormal phenotypes observed by confocal microscopy showing severe myofibrillar degeneration, nuclear morphological defects and revealed additional subcellular defects.

### 2.4 Mutant LamC caused mislocalization of CncC (Nrf2) and increased levels of Ref(2)P (p62)

Abnormal cytoplasmic protein aggregation causes nuclear enrichment of the mammalian nuclear factor erythroid‐related factor 2 (Nrf2) in a mouse model of mutant αB‐crystallin‐induced cardiomyopathy (Kannan et al., 2013; Rajasekaran et al., 2011). Nrf2 and its cytoplasmic binding partner Keap1 function in cellular detoxification and are conserved in Drosophila (Deng & Kerppola, 2013, 2014). In a Drosophila model of noncardiac muscle laminopathies, Cap‐and‐collar C (CncC) [the orthologue of mammalian nuclear erythroid 2‐related factor 2 (Nrf2)] redox transcriptional regulator (Deng & Kerppola, 2013, 2014) accumulated in myonuclei and activated cellular detoxification genes (Dialynas et al., 2015). To determine whether CncC accumulated in this cardiac model of laminopathies, hearts expressing wild‐type and mutant *LamC* were stained with antibody to CncC (Deng & Kerppola, 2013, 2014). Hearts expressing wild‐type *LamC* showed CncC localization within the cytoplasm (Fig. S3). In contrast, hearts expressing mutant *LamC* showed CncC localization within the nucleus and cytoplasm (Fig. S3). Thus, mutant LamC caused nuclear enrichment of CncC.

Translocation of Nrf2 (CncC) into the nucleus in the Drosophila model of noncardiac muscle laminopathies was driven by cytoplasmic protein aggregation (Dialynas et al., 2015). Abundant protein aggregates increased levels of the mammalian autophagy cargo receptor p62 and the Drosophila orthologue Ref(2)P (Nezis et al., 2008). The p62 [Ref(2)P] protein binds Keap1, sequestering it from Nrf2 (CncC) in the cytoplasm, thereby allowing Nrf2 (CncC) to translocate into the nucleus (Dialynas et al., 2015; Jain et al., 2010). Antibodies showed elevated levels of Ref(2)P in protein extracts from hearts expressing mutant LamC via western and antibody dot blots (Figure 4b–d) as well as in immunostained tissues relative to hearts expressing wild‐type *LamC* (Figure 4e, red foci) Thus, cardiac‐specific expression of mutant *LamC* elevated levels of Ref(2)P, promoting CncC nuclear translocation.

Nuclear CncC (Nrf2) is a characteristic of redox imbalance (Deng & Kerppola, 2013, 2014). To determine the redox status of the hearts, reduced (GSH) and oxidized (GSSH) glutathione were measured (Anderson, 1985; Dialynas et al., 2015). The absolute values of both did not show differences between flies expressing wild‐type *LamC* and *G489V* (Fig. S4a); however, the ratio of GSH:GSSG was lower in 3‐week‐old adults expressing *G489V* compared to 3‐week‐old adults expressing wild‐type LamC*,* suggesting an oxidative redox imbalance at this age (Fig. S4a).

### 2.5 Mutant LamC disrupted adipose tissue homeostasis

Mutations in the human *LMNA* gene cause dysregulation of adipose tissue referred to as lipodystrophy (Wiltshire, Hegele, Innes & Brownell, 2013). These individuals experience age‐related loss of subcutaneous fat from their lower body and increased visceral fat in the upper portion of their body. While performing Drosophila heart dissections, we noted an age‐dependent increase in size of the fat bodies of adults expressing mutant *LamC*, relative to controls. Fat body tissue is thought to function similar to vertebrate adipose and liver tissues. The increased adipose tissue was apparent upon staining adults with the lipophilic dye Nile Red (Fowler & Greenspan, 1985; Lee, Bassel‐Duby & Olson, 2014). Nile Red staining showed increased lipid droplet size in 3‐week‐old adults expressing *R205W* and *G489V,* relative to controls (Figures 4f and S4b, upper panels). These cytological findings were supported by quantitative analysis of total triglycerides in adults. One‐day‐old adults with cardiac‐specific expression of wild‐type *LamC* and *G489V* showed no difference in triglyceride levels (Fig. S4b, lower panel). In contrast, 2‐ to 5‐week‐old adults showed significant increases in triglyceride levels. These data demonstrate an age‐dependent increase in total triglycerides.

### 2.6 Suppression of mutant LamC‐induced heart defects by cardiac‐specific upregulation of autophagy and downregulation of CncC (Nrf2)

**Figure 5:**
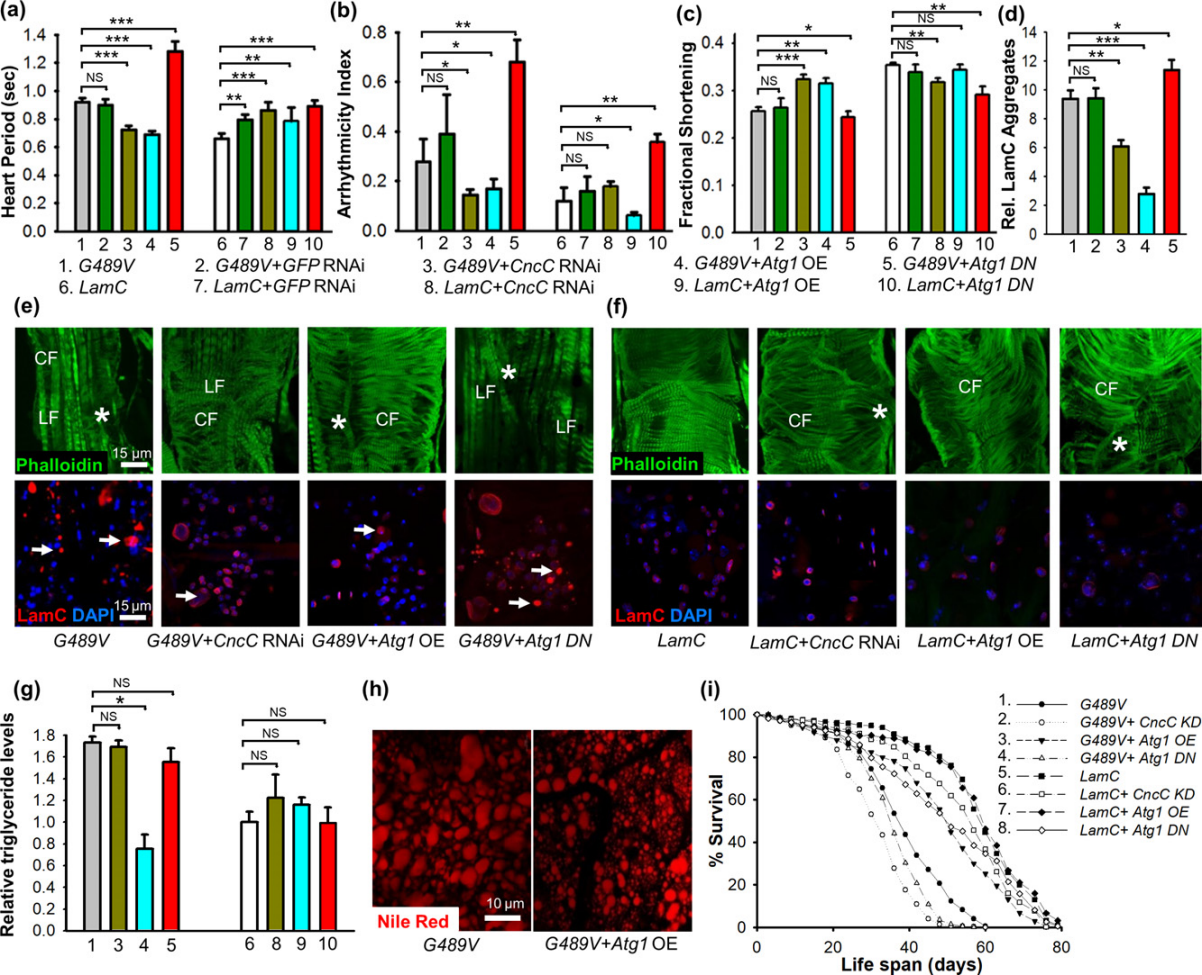
Cardiac‐specific expression of *Atg1* and knockdown of *CncC* suppressed the abnormal cardiac, fat, and aging phenotypes caused by mutant LamC. *Atg1* OE in hearts of 3‐week‐old adults (*n* = 30–70 per genotype) expressing *G489V* suppressed the heart period defects (a), cardiac dysrhythmia (b), and enhanced cardiac performance as represented by fractional shortening (c). In contrast, expression of *Atg1 DN* in hearts of 3‐week‐old adults expressing *G489V* enhanced the cardiac period and cardiac dysrhythmia and caused further deterioration of the heart (a–c). Cardiac‐specific expression of a *CncC* RNAi under the same conditions enhanced cardiac performance and suppressed heart period and cardiac dysrhythmia. An RNAi against *GFP* showed no effect on these cardiac parameters (a–c). These genetic modifiers had little to no effect on the physiology of hearts expressing wild‐type *LamC* (a–c). (d) Suppression of cardiac defects correlated with a reduction in cytoplasmic LamC aggregates. Cardiac‐specific expression of *Atg1* and *CncC* RNAi suppressed the cytoplasmic aggregates, whereas *Atg1 DN* and *GFP* RNAi did not. The relative area of LamC aggregates/total area surveyed in confocal images was plotted. (e) Representative confocal images of the hearts stained with phalloidin (green), antibody against LamC (red), and DAPI (blue) showed a reduction in LamC protein aggregates. Cardiac‐specific of expression of *CncC* RNAi and *Atg1* OE suppressed cytoplasmic LamC aggregates (arrows) and myofibrillar disorganization (*) in 3‐week‐old adults. Cardiac‐specific expression of *Atg1 DN* resulted in increased LamC aggregates and further deterioration of the organization of the actin‐containing myofibrils organization. Contractile circumferential fiber (CF) was missing; however, noncontractile longitudinal fibers (LF) were retained. (f) These genetic manipulations had little to no effect in hearts expressing wild‐type *LamC* (g) Cardiac‐specific *Atg1* OE lowered the levels of total triglycerides. In contrast, *Atg1 DN* and RNAi against *CncC* showed no effect on triglyceride levels. Numbers correspond to the numbered genotypes in panels (a‐c). (h) Representative confocal images of adipose tissue from adults with cardiac‐specific expression of *G489V* and *Atg1* OE stained with Nile Red showed a reduction in lipid droplets compared to the control expressing *G489V* alone. (i) *Atg1* OE lengthened lifespan of adults with cardiac‐specific expression of *G489V*. The lifespan of adults (150–250 female per genotype) was determined for adults of the indicated genotypes. *Atg1* OE suppressed the *G489V*‐induced shortened lifespan, whereas *Atg1 DN* and a *CncC* RNAi did not alter lifespan. The effect of these genetic modulators on lifespan of flies with cardiac‐specific expression of wild‐type *LamC* was used as a control. Statistical significance in A‐D, G, and F is denoted *as follows*: *p <* *.05; **p <* *.01; ***p <* *.001; NS **= not significant

The pathological features exhibited by the Drosophila cardiolaminopathy model included cytoplasmic aggregation of LamC, upregulation of p62, and nuclear enrichment of CncC (Figures 3 and 4). Genetic tools available in Drosophila allow for tissue‐specific over‐expression and RNAi knockdown (KD) of candidate genes that might suppress the cardiac abnormalities (Table S1). Logical candidates include those that increase autophagy (to eliminate cytoplasmic protein aggregates) and block the CncC/Keap1 signaling pathway. Over‐expression (OE) of an *Atg1* transgene encoding a kinase that upregulates autophagy (Neufeld, 2007), suppressed the cardiac defects associated with mutant *LamC* (Figures 5, S5, S6). OE of *Atg1* lowered the heart period, reduced cardiac arrhythmia, and enhanced cardiac performance (shown as fractional shortening) (Figure 5a–c). Importantly, *Atg1* OE in a wild‐type *LamC* genetic background did not alter cardiac dysfunction (Figure 5a–c). Inhibition of Atg1 was achieved by expressing a transgene encoding a dominant negative (DN) Atg1 mutant (Neufeld, 2007), which enhanced the defective cardiac phenotypes caused by expression of *G489V* (Figures 5, S5, S6). It should be noted that expression of *Atg1 DN* in a wild‐type *LamC* genetic background produced mild cardiac phenotypes (Figure 5a–c). Thus, *Atg1* OE suppressed the cardiac phenotypes and *Atg1 DN* enhanced cardiac defects caused by *G489V*.

We next determined whether nuclear enrichment of CncC contributed to the cardiac phenotypes. Cardiac‐specific expression of an RNAi against *CncC* (referred as KD) suppressed the *G489V*‐induced cardiac physiological defects (Figure 5a–c, S5, S6a). It should be noted that cardiac‐specific RNAi against *CncC,* as well as a negative control RNAi against *GFP,* produced subtle cardiac defects in a wild‐type *LamC* background (Figure 5a–c and Fig. S5), suggesting that activation of the RNAi pathway in this context has a minor negative effect on cardiac function. Taken together, these data demonstrate that knockdown of *CncC* suppressed the cardiac defects caused by *G489V*, suggesting that nuclear enrichment of CncC contributes to the cardiac pathology.

To determine the cytological changes that account for the genetic suppression of the heart defects, confocal microscopy was used to image cardiac cells. Cardiac‐specific KD of *CncC* in hearts expressing *G489V* reduced cytoplasmic LamC aggregates, restored nuclear morphology, and promoted organization of actin‐containing myofibrils (Figure 5d–f). Likewise, *Atg1* OE reduced cytoplasmic LamC aggregates, restored nuclear morphology, and promoted actin organization (Figure 5d–f). In contrast, expression of *Atg1 DN* (Neufeld, 2007) increased the number of LamC aggregates, compared to age‐matched adults (Figure 5d, e). Moreover, expression of the *Atg1 DN* in combination with *G489V* resulted in nearly complete loss of contractile circumferential fiber (CF); however, the noncontractile longitudinal fibers remained largely intact (Figure 5d–f). Cardiac‐specific KD of *CncC*,* Atg1* OE, and the *Atg1 DN* resulted in subtle cytological defects in the wild‐type *LamC* background compared to age‐matched controls. Overall, these results suggest that increasing autophagy and reducing CncC/Keap1 redox signaling suppress cytological defects caused by *G489V*.

To test the impact of increasing autophagy and blocking the CncC/Keap1 pathway on adipose tissue homeostasis, both biochemical and cytological approaches were used. The impaired adipose tissue homeostasis caused by *G489V* was suppressed upon cardiac‐specific *Atg1* OE, resulting in decreased triglycerides and smaller lipid droplets (Figure 5g, h). Cardiac‐specific expression of *Atg1 DN* had a minor negative impact on the elevated triglyceride levels (Figure 5g, h). In contrast to the results obtained by modulation of autophagy**,** cardiac‐specific KD of *CncC* did not alter *G489V*‐induced adipose tissue homeostasis defects (Figure 5g). Moreover, cardiac‐specific *Atg1* OE and *CncC* KD did not alter adipose tissue homeostasis in a wild‐type *LamC* background (Figure 5g). Thus, increasing autophagy suppressed both the cardiac phenotypes and adipose tissue phenotypes induced by *G489V*. These results suggest that adipose accumulation might be due to the lack of fat utilization by the heart. However, *CncC* RNAi suppressed the cardiac defects, but not the adipose tissue accumulation, suggesting that the elevated triglycerides are not due to loss of cardiac function per se and might be due to altered systemic lipid homeostasis.

We next determined whether restoration of cardiac function and maintenance of adipose tissue homeostasis impacted the lifespan of adults with cardiac‐specific expression of *G489V*. *Atg1* OE extended the lifespan of adults expressing *G489V*, whereas cardiac‐specific expression of *Atg1 DN* resulted in further shortening of the lifespan (Figure 5i). Cardiac‐specific expression of *CncC* RNAi did not alter the lifespan of adults expressing *G489V*, whereas it shortened the lifespan in adults expressing wild‐type *LamC* (Figure 5i). Taken together, these results indicated that cardiac‐specific expression of *CncC* RNAi suppressed LamC cytoplasmic aggregates, nuclear shape, and cytoskeletal defects, but did not suppress fat accumulation and the shortened lifespan caused by *G489V*. In contrast, *Atg1* OE showed a nearly complete suppression of the abnormal phenotypes and restoration of lifespan.

To determine the generalizability of these findings, we performed similar genetic tests using flies that express the *R205W* transgene. *Atg1* OE suppressed the cardiac dysfunction (Fig. S7a–c), cytoplasmic aggregates (Fig. S7d), and shortened lifespan (Fig. S7e). Collectively, these results are similar to the suppression observed for flies expressing the *G489V* transgene. In contrast, cardiac‐specific KD of *CncC* did not suppress mutant phenotypes caused by *R205W* (Fig. S7a–e). These data suggest that different mutant versions of *LamC* might have different effects on redox homeostasis, a topic for further investigation.

### 2.7 Interplay between autophagy and Nrf2/Keap1 signaling in suppression of cardiac defects

**Figure 6:**
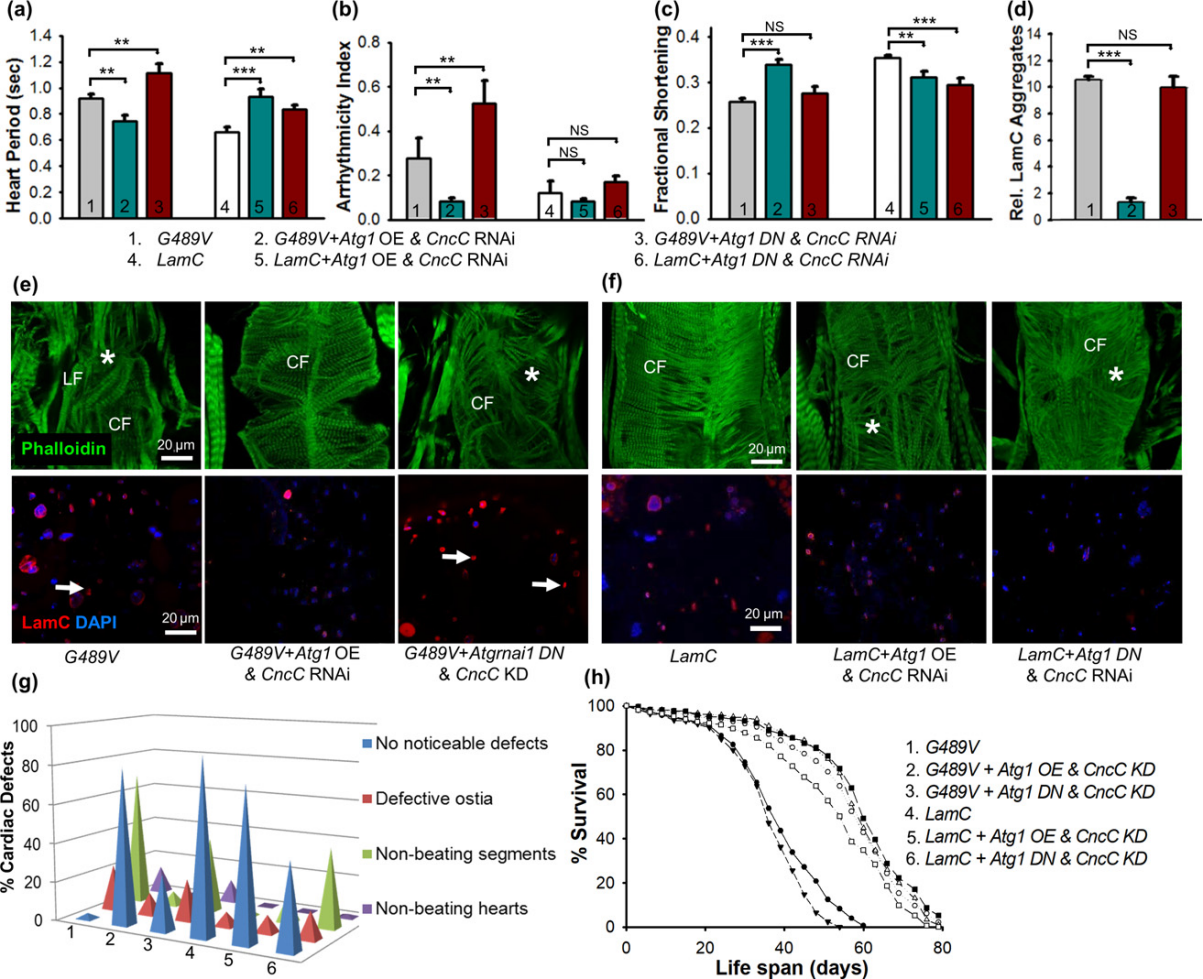
An interplay between autophagy and CncC/Keap1 suppressed cardiac dysfunction caused by mutant LamC. (a–c) The cardiac function of 3‐week‐old flies (*n* = 33–70 for each genotype) expressing wild‐type and mutant *LamC* was compared. Hearts expressing *G489V*,* Atg1* OE, and RNAi against *CncC* showed a reduction in lower heart period (a), cardiac dysrhythmia (b), and enhanced cardiac performance as represented as fractional shortening (c) compared to hearts expressing *G489V* alone. Expression of *G489V* in combination with *Atg1 DN* and a *CncC* RNAi *(KD)* resulted in enhancement of cardiac period and dysrhythmia and increased deterioration compared with hearts expressing *G489V* alone. (d) *Atg1* OE and *CncC* RNAi suppressed the LamC cytoplasmic aggregates. Quantification of the cytoplasmic aggregates was performed by taking the relative average area of the aggregates per the total area of the confocal images surveyed. (e–f) Representative confocal images of hearts from 3‐week‐old adults stained with phalloidin (green), antibodies against LamC (red), and DAPI (blue) showed that simultaneous OE of *Atg1* and a *CncC* RNAi resulted in nearly complete suppression of cytoplasmic LamC aggregation (represented by arrows) and myofibrillar disorganization (represented by *) caused by *G489V*. In contrast, simultaneous expression of *Atg1 DN* and a *CncC* RNAi enhanced the disorganization of the actin‐containing myofibrils, with contractile circumferential fibers (CF) completely disorganized. The impact of this genetic combination resulted in subtle myofibrillar disorganization in the wild‐type *LamC* background, but did not alter nuclear morphology and LamC nuclear envelope localization. (g) Simultaneous cardiac‐specific expression of *Atg1* and a *CncC* RNAi suppressed all of the cardiac parameters analyzed. (h) Simultaneous cardiac‐specific *Atg1* OE with a *CncC* RNAi completely suppressed the *G489V*‐induced shortening of lifespan (150–250 adults were assayed per genotype). Expression of the *Atg1 DN* in combination with a *CncC* RNAi did not rescue the shortened lifespan caused by *G489V*. Statistical significance in A‐D is denoted *as follows*: *p <* *.05; **p <* *.01; ***p <* *0.001; NS **= not significant

To determine whether an interplay exists between autophagy and CncC/Keap1 signaling, *Atg1* (over‐expression and loss of function) was co‐expressed with an RNAi against *CncC* in cardiac tissue expressing wild‐type and mutant *LamC*. *Atg1* OE in combination with *CncC* RNAi (referred to hereafter as the “double treatment”) suppressed the physiological and cytological defects in the heart, restored adipose tissue homeostasis, and sustained lifespan (Figures 6 and S6, S8, S9). Abnormalities in cardiac contractility (Figure 6c), cardiac arrhythmias (Figure 6b), and heart period were suppressed. Additionally, cardiac parameters such as cardiac systolic and diastolic diameters and intervals were more similar to those of hearts expressing wild‐type *LamC* than those without the double treatment (Fig. S8). The cardiac defects of adults expressing *G489V* were enhanced upon cardiac expression of *Atg1 DN* and an RNAi against *CncC* (Figure 6a–c and Fig. S8). Moreover, expression of *Atg1 DN* and an RNAi against *CncC* resulted in altered cardiac physiology (Figure 6a–c and Fig. S8). Taken together, these results support an interplay between autophagy and the CncC/Keap1 pathway in which A*tg1* OE enhances the effects of *CncC* RNAi and profoundly improves cardiac function and lifespan, suggesting modulation of both redox signaling and autophagy as an avenue for therapy.

To determine whether the suppression of cardiac dysfunction observed with the double treatment suppressed the cardiac cellular phenotypes, hearts were stained with antibodies to LamC. There was an absence of cytoplasmic LamC in the suppressed hearts. In addition, the nuclear morphological defects, myofibrillar disorganization, and nuclear enrichment of CncC were suppressed (Figure 6d and S9a, c). In contrast, simultaneous cardiac‐specific expression of *Atg1 DN* and a *CncC* RNAi did not suppress LamC cytoplasmic aggregates, nuclear defects, and myofibrillar disorganization (Figure 6d). In addition, enrichment of CncC was enhanced (S9D). In controls expressing wild‐type *LamC*, the double treatment caused minor myofibrillar disorganization, but had no apparent effect on LamC localization and nuclear morphology (Figure 6f). Collectively, these findings demonstrate that upregulation of autophagy and blocking the CncC/Keap1 pathway ameliorates the cardiac cellular defects caused by mutant LamC.

Simultaneous manipulation of autophagy and the CncC/Keap1 pathway was also tested for effects on adipose tissue homeostasis. The double treatment reduced total triglyceride levels in adults expressing *G489V* (Fig. S9e). In contrast, simultaneous expression of *Atg1 DN* and *CncC* RNAi did not alter the elevated triglyceride levels (Fig. S9e). Furthermore, these genetic combinations did not significantly change triglyceride levels in the *LamC* background (Fig. S9e). We reason that the suppressive effect of the double treatment on triglycerides is likely through increased autophagy as a similar effect was observed with *Atg1* OE alone.

We next determined whether restoration of cardiac function and maintenance of adipose tissue homeostasis impacted the lifespan of *G489V*‐expressing adults (Figure 6h). The double treatment completely restored lifespan (Figure 6h). In contrast, simultaneous expression of *Atg1 DN* and an RNAi against *CncC* caused further reduction in the lifespan compared to flies expressing *G489V* alone (Figure 6h). As a control, the double treatment had little effect on the lifespan of adults with cardiac‐specific expression of wild‐type *LamC*. However, cardiac‐specific expression of *Atg1 DN*,* CncC* RNAi, and wild‐type *LamC* reduced lifespan compared to *LamC* alone. Taken together, these findings demonstrated that simultaneous over‐expression of *Atg1* in combination with expression of *CncC* RNAi suppressed cytoplasmic aggregation of LamC, restored cardiac contractility, and improved lifespan.

## 3 DISCUSSION

Mutations in the human *LMNA* gene are associated with a collection of diseases called laminopathies in which the most common manifestation is progressive cardiac disease (Brayson & Shanahan, 2017; Heller et al., 2017; Marian, 2017; Naetar, Ferraioli & Foisner, 2017). We have generated *Drosophila melanogaster* models of age‐dependent cardiac dysfunction. In these models, mutations synonymous with those causing disease in humans were introduced into Drosophila *LamC*. Cardiac‐specific expression of mutant *LamC* resulted in (1) cardiac contractility, conduction, and physiological defects, (2) abnormal nuclear envelope morphology, (3) cytoplasmic LamC aggregation, (4) nuclear enrichment of the redox transcriptional regulator CncC (mammalian Nrf2), (5) and upregulation of autophagy cargo receptor Ref(2)P (mammalian p62) (Figures 1, 2, 3, 4, S3). These cardiac defects were enhanced with age (Figures 2 and S2) and accompanied by increased adipose tissue in the adult fat bodies (Figure 4f) and a shortened lifespan (Figure 1d, e).

**Figure 7:**
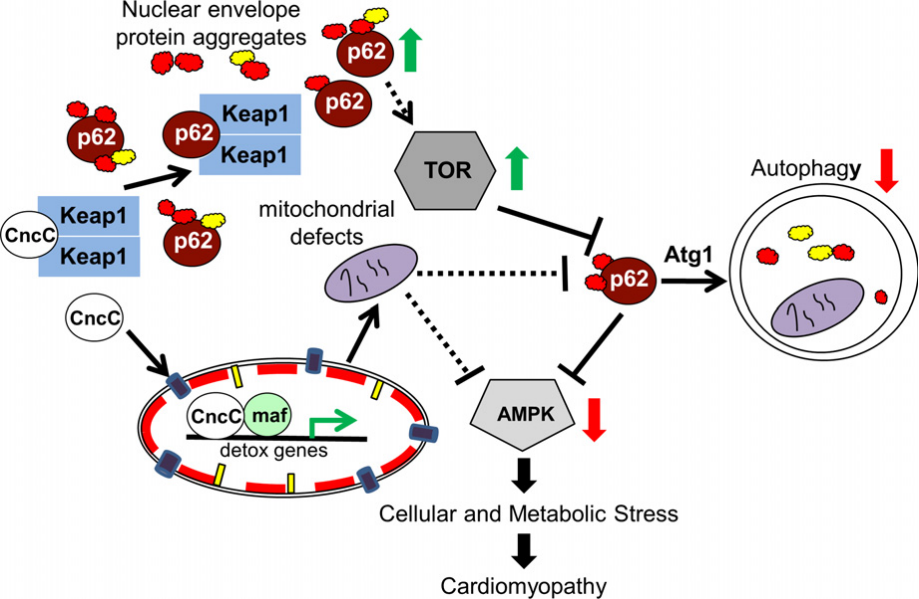
Model for the interactions between the autophagy and CncC/Keap1 signaling pathway in mutant lamin‐induced cardiac disease. Cellular and metabolic stress is triggered by the abnormal aggregation of nuclear envelope proteins in the cytoplasm (red and yellow circles with irregular margins), leading to increased levels of Ref(2)P (p62). Sequestration of Keap1 by Ref(2)P allows CncC (Nrf2) to enter the nucleus and activate antioxidant genes. Exhaustion of the antioxidant system is hypothesized to cause redox imbalance, potentially through mitochondrial dysfunction and dysregulation of energy homeostasis. Upregulation of Ref(2)P leads to activation of TOR, inactivation of autophagy, and inactivation of AMPK. Simultaneous over‐expression of *Atg1* and inhibition of CncC suppressed cytoplasmic aggregation of LamC and restored cardiac contractility and lifespan

To understand the mechanistic basis of cardiolaminopathy and identify genetic suppressors, we took advantage of powerful genetic tools available in Drosophila. The presence of cytoplasmic LamC aggregates prompted us to determine whether increasing autophagy would suppress the cardiac defects. Cardiac‐specific upregulation of autophagy (*Atg1* OE) suppressed *G489V*‐induced cardiac defects (Figures 5 and S5–S6). Consistent with this, decreased autophagy due to expression of *Atg1 DN* resulted in enhanced deterioration of *G489V*‐induced cardiac dysfunction (Figures 5 and S5). Interestingly, cardiac‐specific *Atg5* OE and *Atg8a* OE, two factors that also promote autophagy, showed little to no suppression of *G489V*‐induced heart dysfunction (Table S1), suggesting that *Atg1* might be rate limiting in this context. Our findings are consistent with studies in mouse laminopathy models in which rapamycin and temsirolimus had beneficial effects on heart and skeletal muscle through inhibition of AKT/mTOR signaling (Choi et al., 2012; Ramos et al., 2012). Our findings are depicted in a model (Figure 7) in which cytoplasmic aggregation of mutant LamC results in upregulation of p62, which in turn inhibits autophagy via activation of TOR and inactivation of AMPK (Mihaylova & Shaw, 2011). AMPK inactivation leads to the activation of PI3K/Akt/mTOR pathway and inhibition of autophagy (Porta, Paglino & Mosca, 2014) *Atg1* OE promoted clearance of the LamC aggregates and restored proteostasis in these Drosophila models. Thus, our data suggest that mutant LamC reduces autophagy, resulting in impairment of cellular proteostasis that leads to cardiac dysfunction.

Cardiac‐specific expression of mutant *LamC* altered CncC subcellular localization (Fig. S3). Previously, Drosophila larval body wall muscles expressing *G489V* were shown to experience reductive stress, an atypical redox state characterized by high levels of reduced glutathione and NADPH, and upregulation CncC target genes (Dialynas et al., 2015). Cardiac‐specific *CncC* RNAi in the wild‐type *LamC* background did not produce major cardiac defects (Figures 5 and S5). Consistent with this, Nrf2 deficiency in mice does not compromise cardiac and skeletal muscle performance (Kannan et al., 2013; Rajasekaran et al., 2011). Cardiac‐specific *CncC* RNAi suppressed *G489V‐*induced cardiac dysfunction and reduced cytoplasmic LamC aggregation, but not *R205W*‐induced defects (Figures 5, S5‐S6, S7). However, cardiac‐specific RNAi against *CncC* did not affect G*489V*‐induced adipose tissue accumulation and lifespan shortening**.** Similar to the nuclear enrichment of CncC in hearts expressing *G489V* (Fig. S3), human muscle biopsy tissue from an individual with a point mutation in the *LMNA* gene that results in G449V (analogous to Drosophila G489V) showed nuclear enrichment of Nrf2 (Dialynas et al., 2015). Disruption of Nrf2/Keap1 signaling has also been reported for Hutchinson–Gilford progeria, an early‐onset aging disease caused by mutations in *LMNA* (Kubben et al., 2016). In this case, however, the thickened nuclear lamina traps Nrf2 at the nuclear envelope that results in a failure to activate Nrf2 target genes, leading to oxidative stress (Kubben et al., 2016). In our studies, we observed CncC nuclear enrichment; however, a redox imbalance was not readily observed at the three‐time points investigated (Fig. S4b). This might indicate that there is a window of time in disease progression in which redox imbalance occurs and that mechanisms are in place to re‐establish homeostasis.

It has been postulated that there is cross‐talk between autophagy and Nrf2/Keap1 signaling (Jain et al., 2010; Stepkowski & Kruszewski, 2011). We tested for this by manipulating autophagy and CncC (Nrf2) alone and in combination. *CncC* RNAi suppressed the cardiac defects caused by *G489V,* but not the lipid accumulation and lifespan shortening, suggesting the latter two phenotypes are not specifically due to loss of cardiac function (Figures 5 and S5–S6). In contrast, *Atg1* OE suppressed the cardiac and adipose tissue defects and lengthened the lifespan. The double treatment (simultaneous *Atg1* OE and RNAi knockdown of *CncC*) gave the most robust suppression of the mutant phenotypes (Figures 6 and S6–S9) and completely restored the lifespan (Figure 6h). Interestingly, *Atg1 DN* and RNAi knockdown of *CncC* simultaneously did not further deteriorate or improve the mutant phenotypes. Taken together, these data suggest that autophagy plays a key role in suppression of the *G498V*‐induced phenotypes and that knockdown on *CncC* enhances this suppression.

Our findings support a model whereby autophagy and Nrf2 signaling are central to cardiac health (Figure 7). We propose that cytoplasmic aggregation of LamC increases levels of Ref(2)P (p62), which competitively binds to Keap1 (Dialynas et al., 2015; Jain et al., 2010), resulting in CncC (Nrf2) translocation to the nucleus. Inside the nucleus, Nrf2 regulates genes involved in detoxification (Dialynas et al., 2015; Jain et al., 2010). Continued expression of antioxidant genes results in the disruption of redox homeostasis, defective mitochondria, and dysregulation of energy homeostasis/energy sensor such as AMPK and its downstream targets. Simultaneously, upregulation of Ref(2)P (p62) causes inhibition of autophagy via activation of TOR, which leads to the inactivation of AMPK (Mihaylova & Shaw, 2011). AMPK inactivation in combination with activation of the TOR pathway causes cellular and metabolic stress that leads to cardiomyopathy. In support of our model, transcriptomics data from muscle tissue of an individual with muscular dystrophy expressing Lamin A/C G449V (analogous to Drosophila G489V) showed (1) upregulation of transcripts from Nrf2 target genes, (2) upregulation of genes encoding subunits of the mTOR complex, and (3) downregulation of *AMPK* (unpublished data), further demonstrating relevance of the Drosophila model for providing insights on human pathology.

## 4 EXPERIMENTAL PROCEDURES

Due to space limitation, experimental procedures are described in the Supplementary section.

## CONFLICT OF INTEREST

None declared.

## AUTHOR CONTRIBUTIONS

GCM and LLW designed the project, conducted experiments, evaluated data, and prepared the manuscript; SB, AST, MTO, GHY, DEC, SC, and MN conducted experiments and evaluated data. All authors have reviewed the manuscript.
