# Supplementary figures and images for: Increasing autophagy and blocking Nrf2 suppress laminopathy‐induced age‐dependent cardiac dysfunction and shortened lifespan
Source: Aging Cell. 2018 Mar 25;17(3):e12747. doi: 10.1111/acel.12747 (PMC5946079; doi:10.1111/acel.12747)

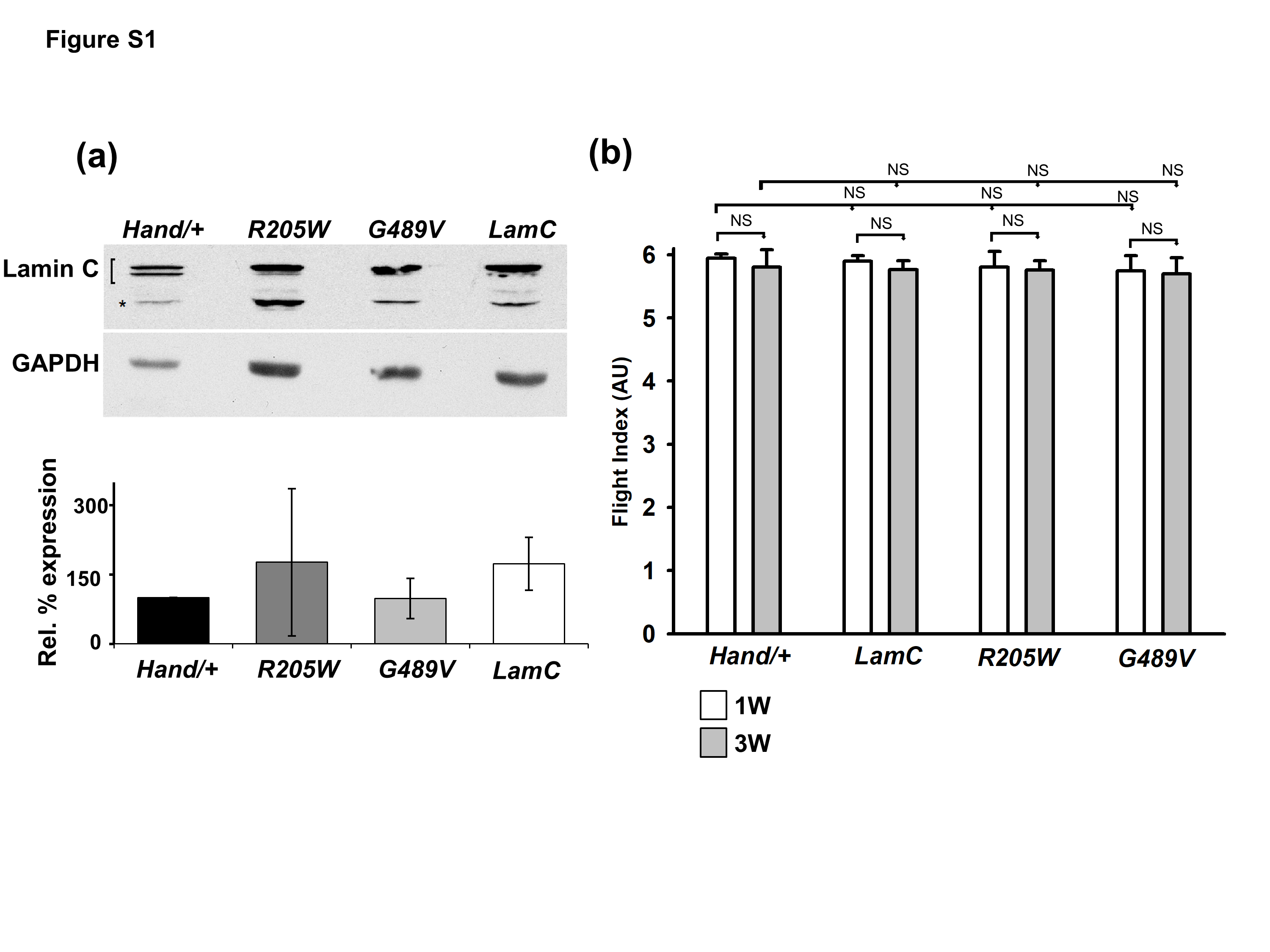

Supplement: Supplementary file 1 [file ACEL-17-e12747-s001.TIF]

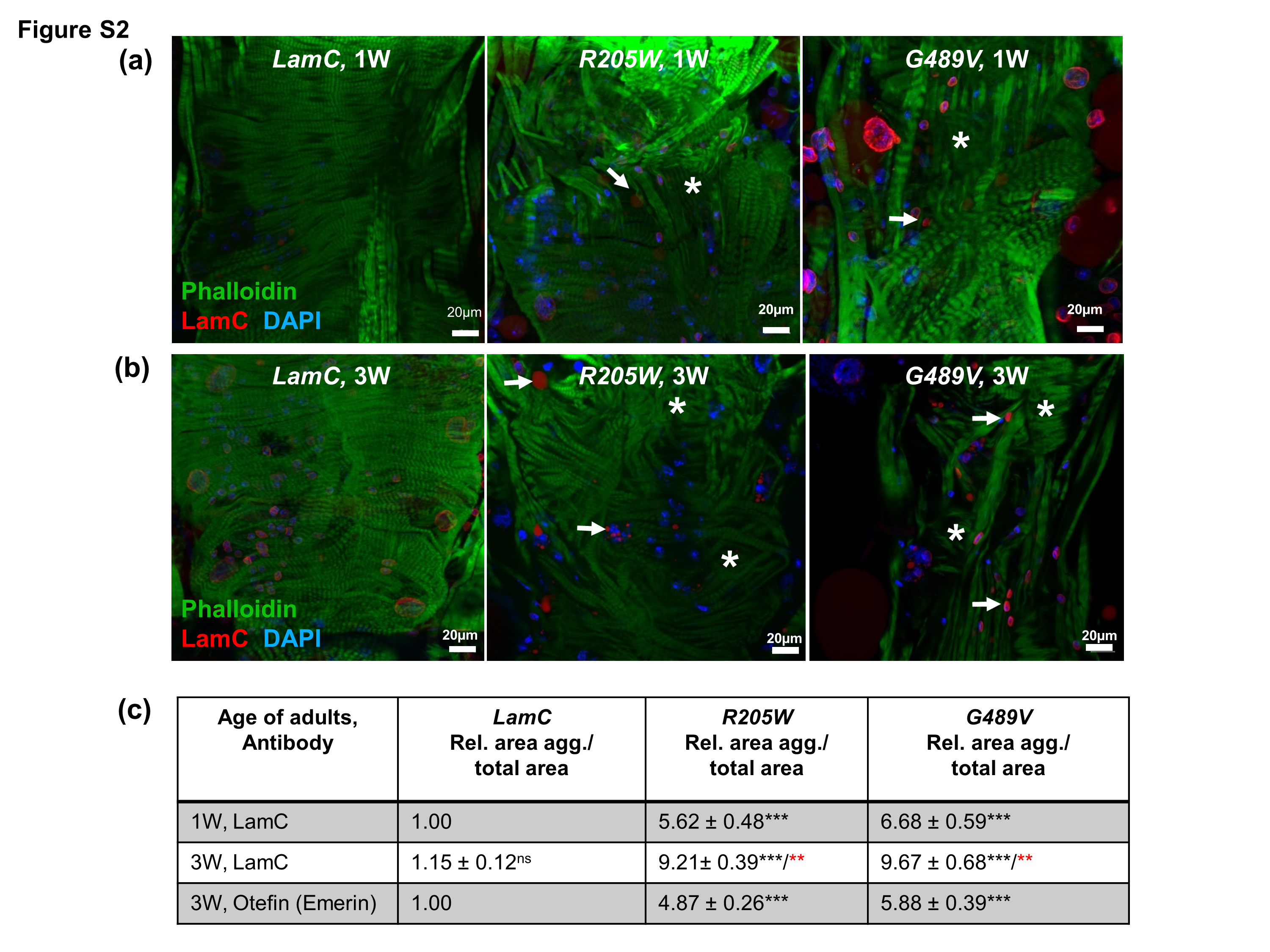

Supplement: Supplementary file 2 [file ACEL-17-e12747-s002.TIF]

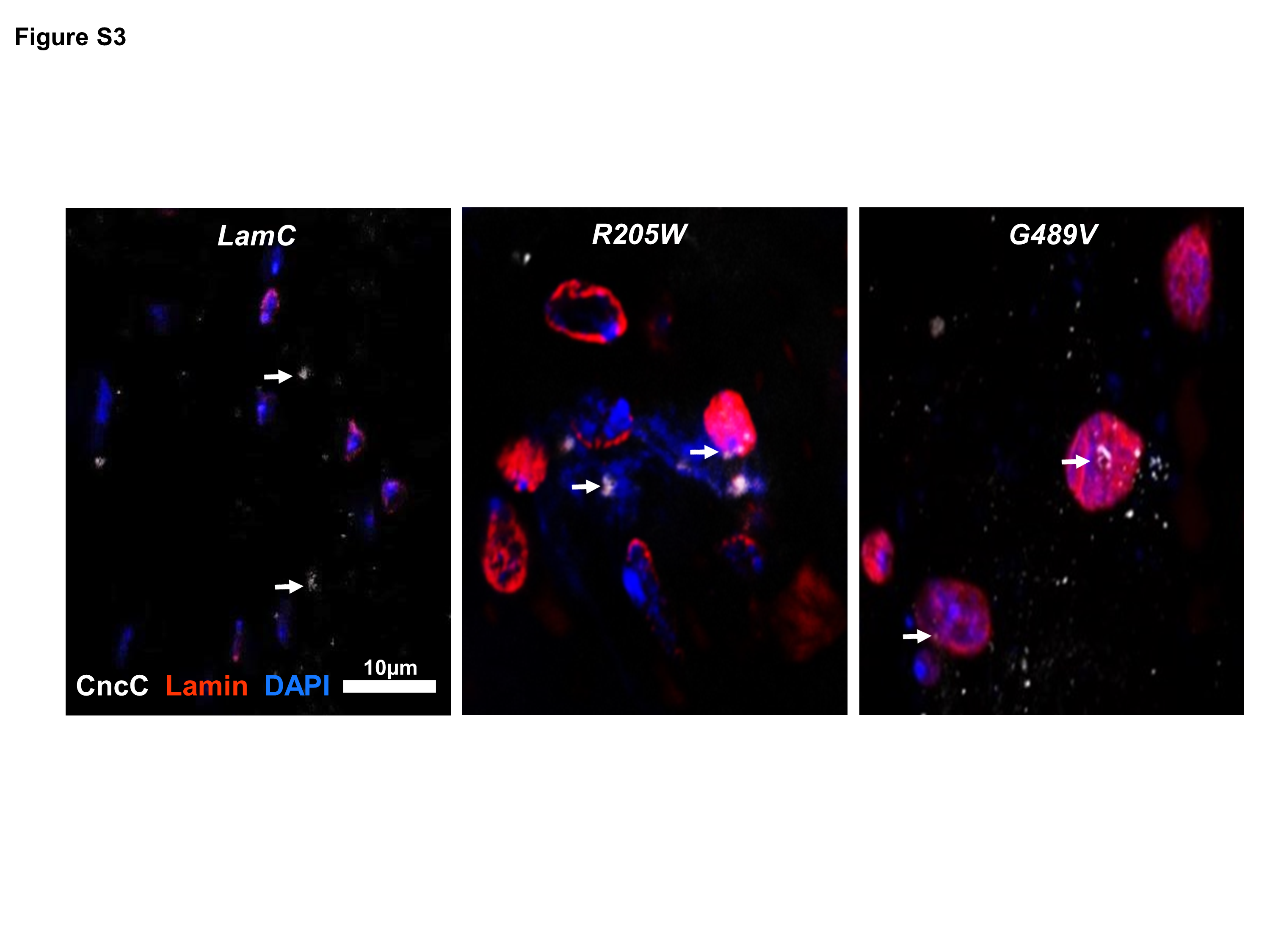

Supplement: Supplementary file 3 [file ACEL-17-e12747-s003.TIF]

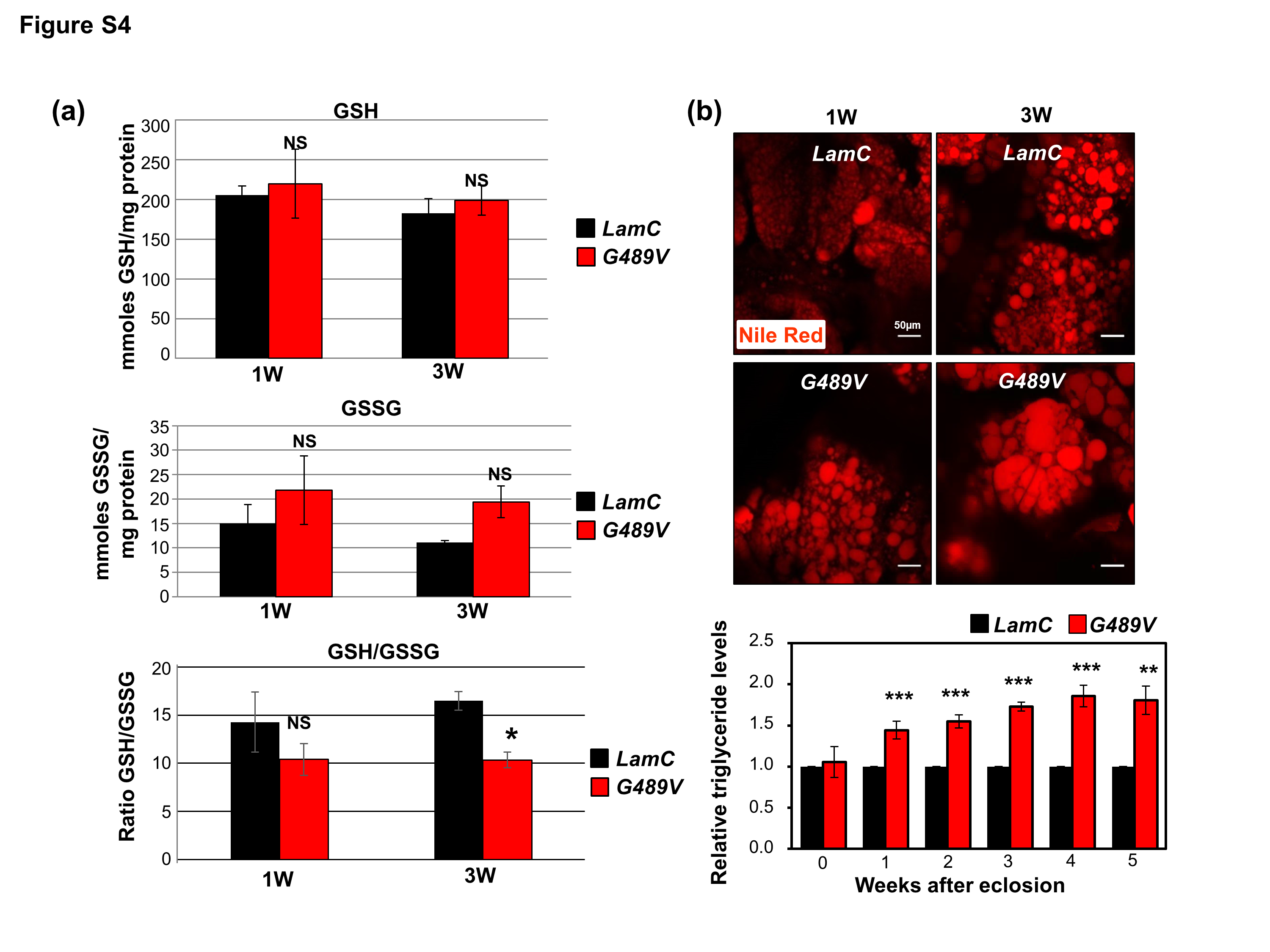

Supplement: Supplementary file 4 [file ACEL-17-e12747-s004.TIF]

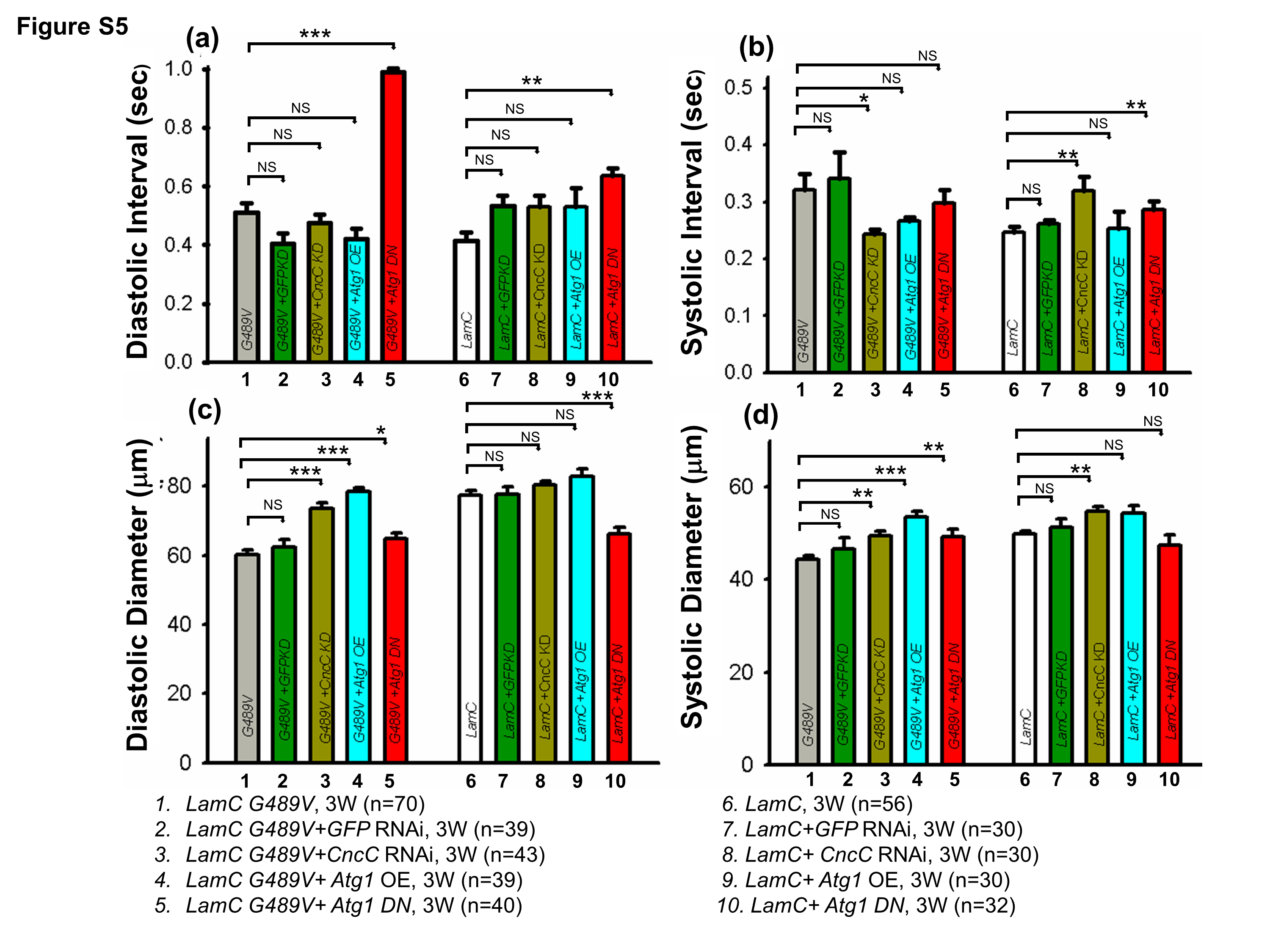

Supplement: Supplementary file 5 [file ACEL-17-e12747-s005.TIF]

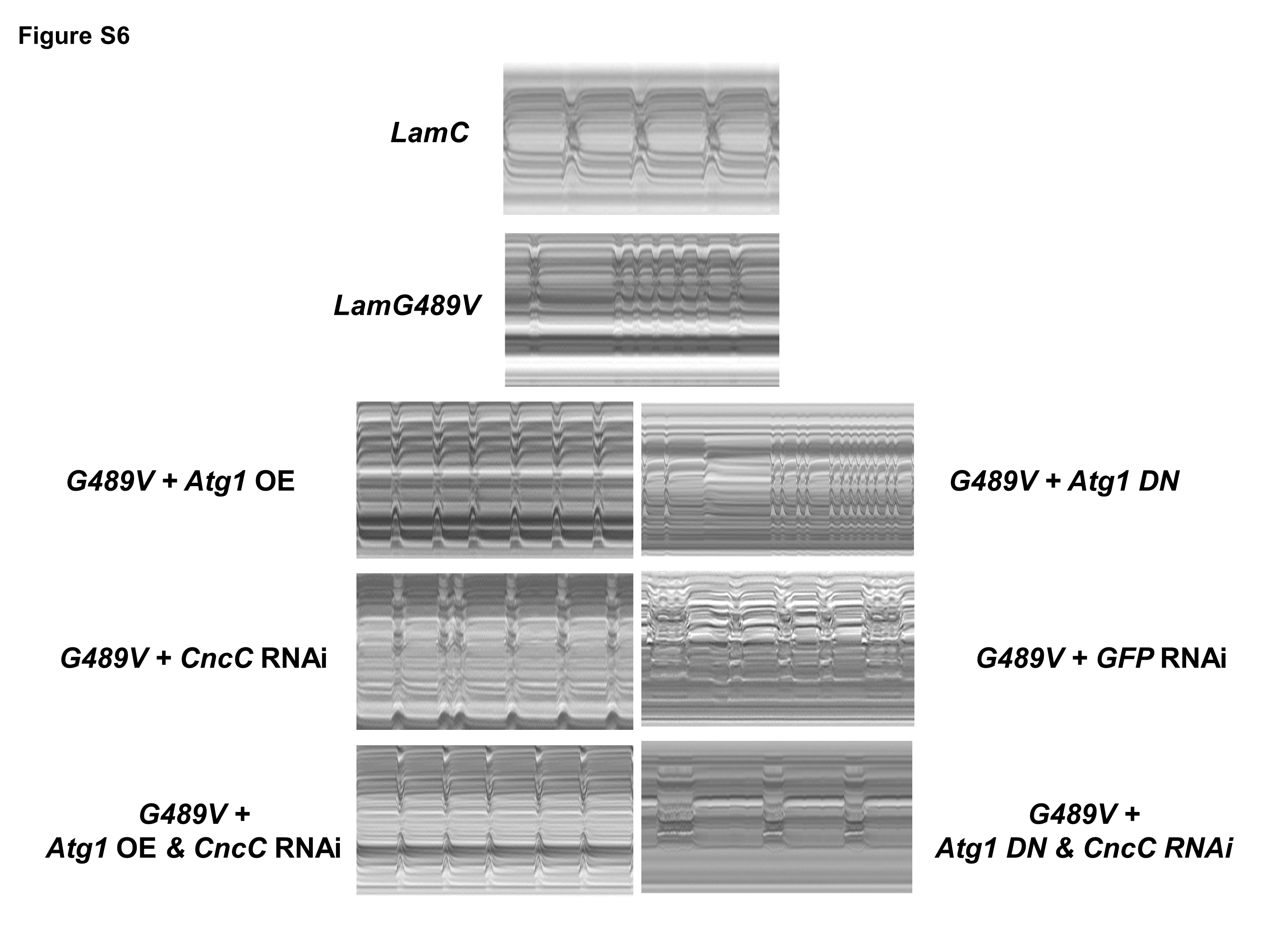

Supplement: Supplementary file 6 [file ACEL-17-e12747-s006.TIF]

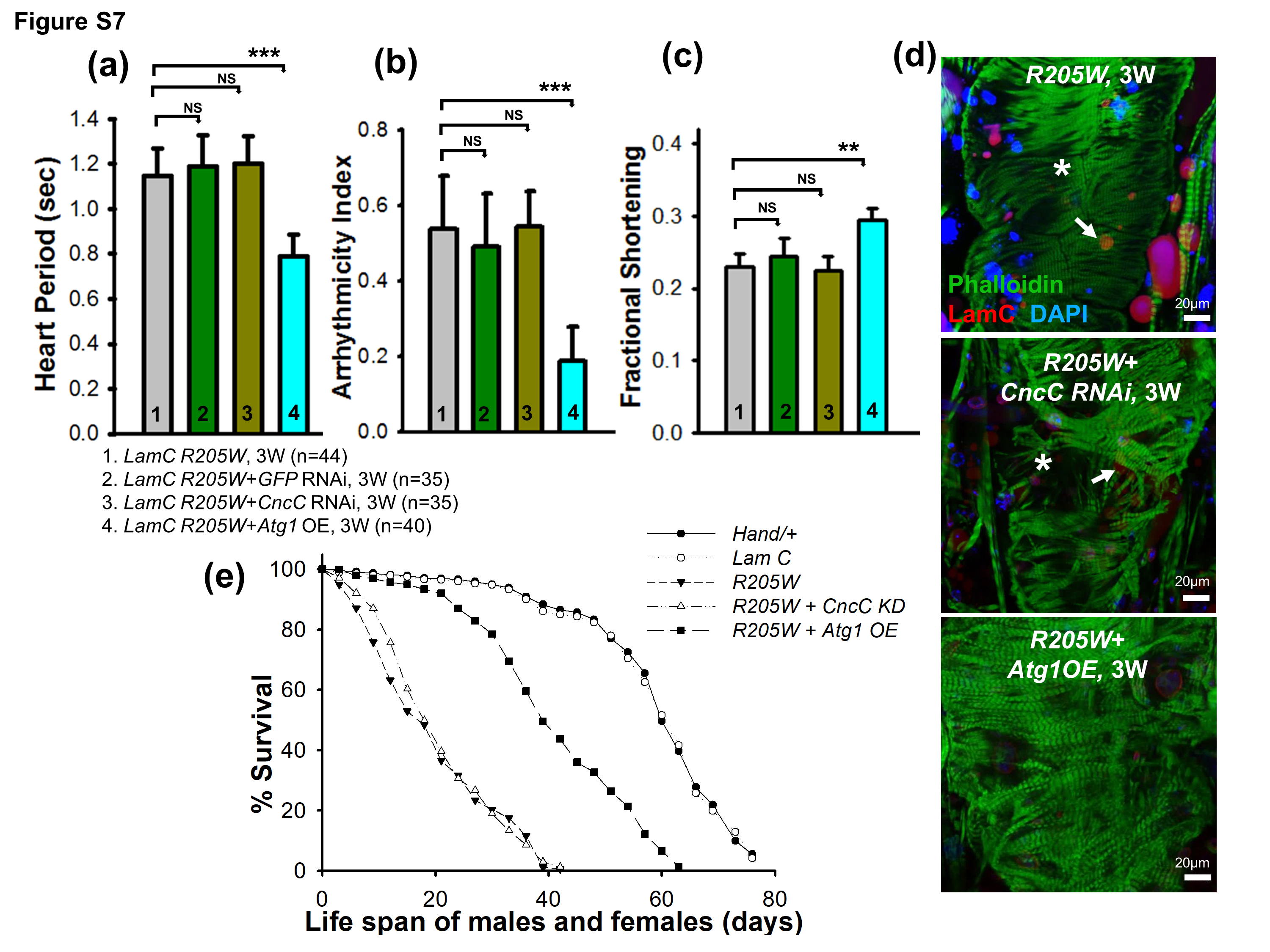

Supplement: Supplementary file 7 [file ACEL-17-e12747-s007.TIF]

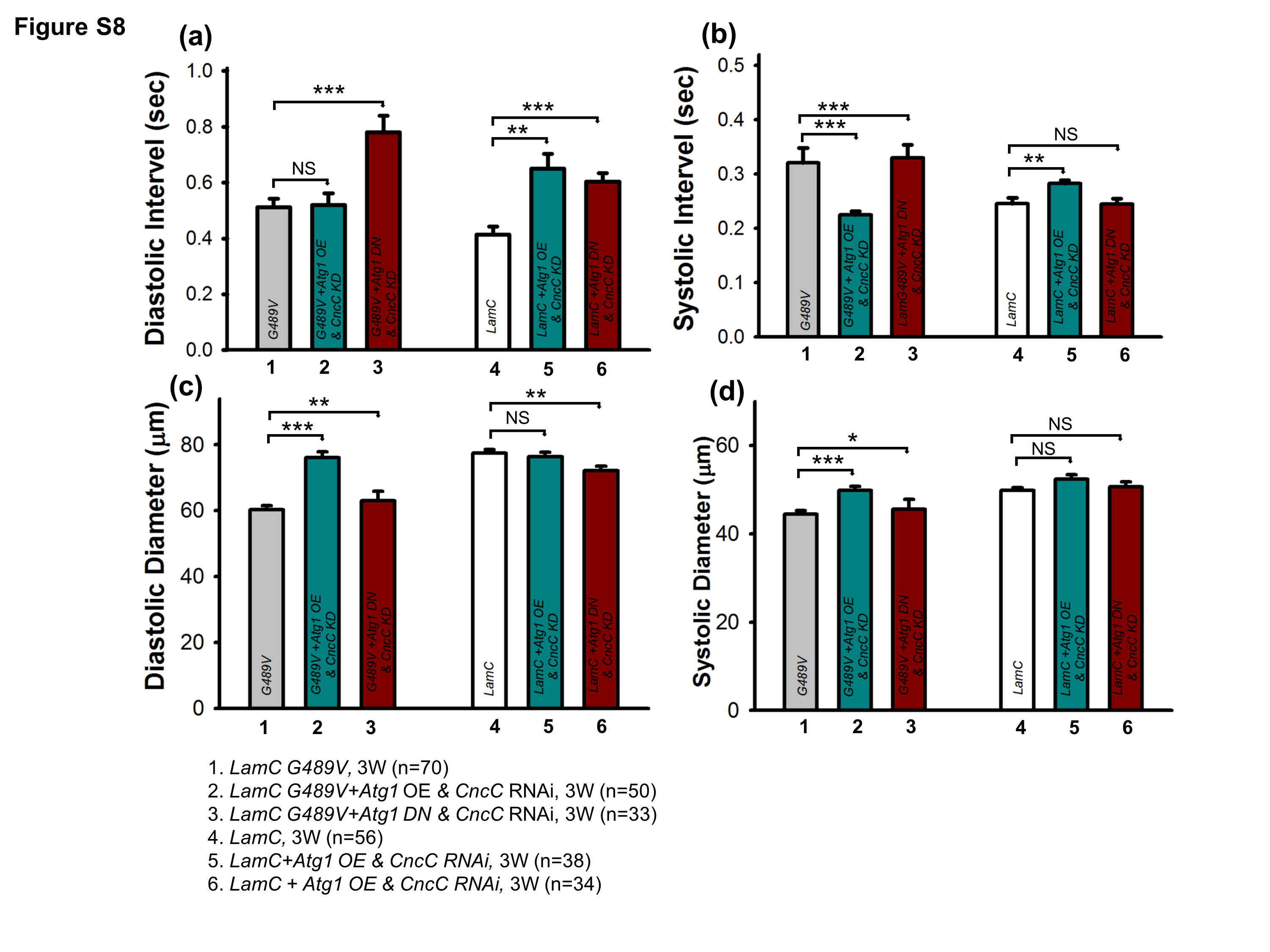

Supplement: Supplementary file 8 [file ACEL-17-e12747-s008.TIF]

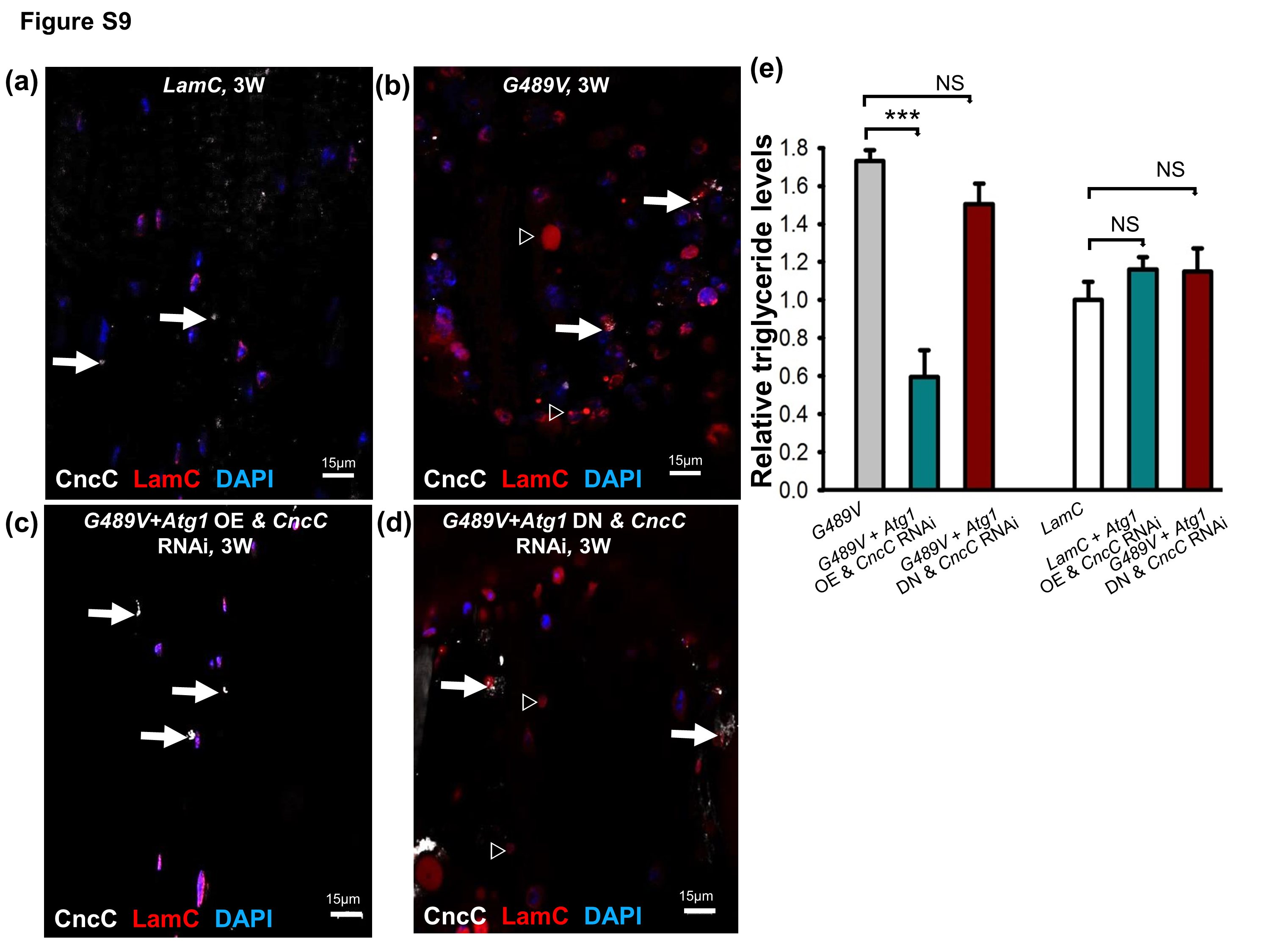

Supplement: Supplementary file 9 [file ACEL-17-e12747-s009.TIF]
