# Supplementary material for: Increasing autophagy and blocking Nrf2 suppress laminopathy‐induced age‐dependent cardiac dysfunction and shortened lifespan
Source: Aging Cell. 2018 Mar 25;17(3):e12747. doi: 10.1111/acel.12747 (PMC5946079; doi:10.1111/acel.12747)
Supplement: Supplementary file 10 [file ACEL-17-e12747-s010.docx]

### Table S1 Summary of genetic modifiers tested for effects on *LamG489V-*induced mutant phenotypes in three-week-old aduls

| **Genotypes (*Hand-Gal4* driven *UAS-Transgenic*)** | **Cardiac physiology** | **Myofibril organization** | **Lamin aggregates and nuclear morphology** | **CncC (Nrf2) localization** | **Ref2P (P62) levels** | **Lipid profile (Nile-Red) staining** | **Life-span** |
| --- | --- | --- | --- | --- | --- | --- | --- |
| *LamC* | Normal | Organized | Absent & normal | Cytoplasmic | Normal | Normal | Normal |
| *LamG489V* | Restricted heart | Severe disorganization | Present and large | Nuclear & cytoplasmic | Upregulated | Significantly large | Shorten |
| *LamC +GFP KD* | normal | organized | Absent & Normal | Not tested | Not tested | Not tested | Normal |
| *LamG489V +GFP KD* | Restricted heart | More  disorganization | Present and large | Not tested | Not tested | Not tested | Shorten |
| *LamC + CncC KD , CG43286* | Subtle cardiac phenotype | Subtle muscle disorganization | Normal | Not tested | No change | Not affected | Subtle  shorten |
| *LamG489V + CncC (Nrf2) KD, CG43286* | Improved | More organized | Suppressed | Not tested | Upregulated | Unchanged | Not improved |
| *LamC +Keap-1 KD (CG3962)* | Subtle cardiac phenotype | Subtle muscle disorganization | Normal | No change | No change | Not affected | Subtle  shorten |
| *LamG489V + Keap-1 KD (CG3962)* | Deteriorated | More disorganized | More aggregate and large nuclei | Nuclear & cytoplasmic | Upregulated | Unchanged | Further shorten |
| *LamC +GSTD4 (CG11512)* | Subtle cardiac phenotype | Subtle muscle disorganization | Normal | Not tested | No change | Not affected | Subtle  shorten |
| *LamG489V + GSTD4 (CG11512)* | Subtle improved | More organized | Suppressed | Not tested | Upregulated | Unchanged | Not improved |
| *LamC +GSTD9 (CG10091)* | Subtle cardiac phenotype | Subtle muscle disorganization | Normal | Not tested | Not tested | Not affected | Subtle  shorten |
| *LamG489V + GSTD9 (CG10091)* | Improved cardiac physiology | More organized | Suppressed | Not tested | Not tested | Unchanged | Not improved |
| *LamC +Glutathione peroxidase (CG12013)* | Subtle cardiac phenotype | Subtle muscle disorganization | Normal | Not tested | Not tested | Not affected | Subtle  shorten |
| *LamG489V + Glutathione peroxidase (CG12013)* | Subtle improved | More organized | Suppressed | Not tested | Not tested | Unchanged | Not improved |
| *LamC +Thioredoxin reductase (CG2151)* | Subtle cardiac phenotype | Subtle muscle disorganization | Normal | Not tested | Not tested | Not affected | Subtle  shorten |
| *LamG489V + Thioredoxin reductase (CG2151)* | Subtle improved | More organized | Suppressed | Not tested | Not tested | Unchanged | Not improved |
| *LamC +Atg-1 Overexpression* | Subtle cardiac phenotype | Subtle muscle disorganization | Normal | Cytoplasmic | unchanged | Not affected | Not affected |
| *LamG489V+ Atg-1 Overexpression (OE)* | Much improved | More organized | Suppressed | Cytoplasmic | Suppressed | Uuppressed | Much improved |
| *LamC +ATG-1 Dominant negative* | Subtle restricted heart | disorganized | normal | Cytoplasmic | Unchanged | Not affected | Reduced |
| *LamG489V+ ATG-1 Dominant negative (DN)* | Deteriorated | More disorganized | More aggregate and large nuclei | Nuclear & cytoplasmic | Unchanged | Not affected | Further shorten |
| *LamC +Atg-5 Overexpression* | Normal | Normal | Normal | Not tested | Not tested | Not affected | Not affected |
| *LamG489V+ Atg-5 Overexpression* | Subtle improved | Subtle organized | Some suppression | Not tested | Not tested | Some suppression | Some improvement |
| *LamC +Atg-8a Overexpression* | Normal | Normal | Normal | Not tested | Not tested | Not affected | Not affected |
| *LamG489V+ Atg-8a Overexpression* | Subtle improved | Subtle organized | Some suppression | Not tested | Not tested | Some suppression | Some improvement |
| *LamC +Atg-1 OE + CncC KD* | Subtle cardiac phenotype | Subtle muscle disorganization | Normal | Cytoplasmic | Unchanged | unchanged | unchanged |
| *LamG489V+ Atg-1 OE & CncC KD* | Completely improved | Organized myofibrils | No aggregates and normal nuclei | Cytoplasmic and nuclear | Suppressed | Complete suppression | Completely rescued |
| *LamC +Atg-1 DN + CncC KD* | Subtle cardiac phenotype | Subtle muscle disorganization | More aggregates | Cytoplasmic | unchanged | Unchanged | Reduced |
| *LamG489V+ Atg-1 DN & CncC KD* | Deteriorated | More disorganized | More aggregate and large nuclei | Mostly nuclear | unchanged | Unchanged | Further shorten |
