# Supplementary material for: Increasing autophagy and blocking Nrf2 suppress laminopathy‐induced age‐dependent cardiac dysfunction and shortened lifespan
Source: Aging Cell. 2018 Mar 25;17(3):e12747. doi: 10.1111/acel.12747 (PMC5946079; doi:10.1111/acel.12747)
Supplement: Supplementary file 11 [file ACEL-17-e12747-s011.docx]

### Supplementary Information:

### Experimental Procedures:

### Drosophila Stocks

### Drosophila stocks were cultured on standard agar media at 25°C ([Melkani et al., 2013](#_ENREF_12)). Stocks with the wild type *LamC* and mutant (*G489V*) lamin transgenes were previously described ([Dialynas et al., 2012](#_ENREF_4); [Dialynas et al., 2015](#_ENREF_5)). For the generation of *R205W* transgenic stock, a full length *LamC* cDNA was amplified by PCR from 18- to 21- hour embryonic RNA (Clonetech, Palo Alto, CA). The cDNA was cloned into pCR2.1-TOPO (Invitrogen, San Diego, CA) and used as a template for site-directed mutagenesis (Quick Change Kit, Qiagen) using following primer set: GCGGAGACGCTGGCCTGGGTCGATCTGGAGAACC (forward) and GGTTCTCCAGATCGACCCAGGCCAGCGTCTCCGC (reverse). The cDNA possessing the mutation was cloned into the pUAST P-element transformation vector and transformants were generated using standard procedures as previously reported ([Dialynas et al., 2012](#_ENREF_4); [Dialynas et al., 2015](#_ENREF_5)). *Hand*-*Gal4* driver was a gift from Eric Olson ([Han & Olson, 2005](#_ENREF_9)). The Atg1 stocks were obtained from the Bloomington Stock Center. UAS-RNAi stocks for knock-down of *CncC (Nrf2),* glutathione metabolism genes and *GFP* ([Dietzl et al., 2007](#_ENREF_6)) were obtained from the Vienna Drosophila RNAi Center (VDRC).

**Cardiac-specific expression and genetic modulation**

The GAL4-UAS system ([Duffy, 2002](#_ENREF_7)) was used to drive expression of wild type and mutant *LamC* transgenes in heart tissue using the *Hand-Gal4* driver. Adult flies possessing UAS transgenes encoding wild type and mutant forms of *LamC* were crossed to Hand*-Gal4* flies and incubated at 25°C throughout development. Adult male and female F1 progeny were separated according to sex and allowed to age, with a new food source supplied every three days prior to assays of cardiac function. Age-matched adults from the host stock *w^1118^* were crossed with *Hand-Gal4* as a control.

Standard genetic and transgenic methodologies were used to co-express Atg1 and CncC-related transgenes in the heart using a *Hand-Gal4* driver and balancer chromosomes. A recombinant stock possessing the *Hand-Gal4* driver and *LamC G489V* was made by crossing w; *Hand/Hand; Tm2/Tm6B* with w; *CyO/Br; G489V/G489V*. F1 adults *(*w*; Hand/CyO; G489V/Tm6B*) were collected and self-crossed to obtain the recombinant line *(*w*; Hand/Hand; G489V/G489V*). For over-expression of Atg1*,* adults possessing the *Atg1* transgene *(*w*; Atg1 OE /Atg1 OE; +)* were crossed to adults possessing the *Hand-Gal4* driver and the mutant *LamC* transgene (*Hand/Hand; G489V/G489V)*. The resulting F1 generation (w*; Hand/Atg1 OE; G489V/+)* was used for analyses. A similar strategy was used for the *Atg1 DN* transgene (2^nd^ chromosome insert), the *CncC* RNAi stock (3^rd^ chromosome insert) and additional transgenes tested.

For the simultaneous expression of Atg1 (OE or DN) and an RNAi against *CncC*, recombinant stocks w*; Atg1 OE/Atg1 OE; CncC RNAi/CncC RNAi and* w*; Atg1 DN/Atg1 DN; CncC RNAi/CncC RNAi* were generated using standard crosses that incorporated balancer chromosomes. Adults from these stocks were separately crossed to w*;* *Hand/Hand; G489V /G489V* adults to obtain following progeny: w*; Atg1 OE/Hand; CncC RNAi/G489V* and w*; Atg1 DN/Hand; CncC RNAi/G489V.* A similar genetic approach was used to study effects on R205W and wild type *LamC*.

**Cardiac physiological analyses** **of semi-intact Drosophila hearts**

Semi-intact hearts were prepared as described ([Fink et al., 2009](#_ENREF_8); [Melkani et al., 2013](#_ENREF_12)). Direct immersion optics was used in conjunction with a digital high-speed camera (up to 200 frame/sec, Hamamatsu EM-CCD) to record 30 second movies of beating hearts; images were captured using HC Image (Hamamatsu Corp.). Cardiac function was analyzed from the high-speed movies using semi-automatic optical heartbeat analysis software that quantifies heart period, diastolic and systolic diameters, diastolic and systolic intervals, cardiac rhythmicity, fractional shortening and produced the M-mode records ([Fink et al., 2009](#_ENREF_8); [Melkani et al., 2013](#_ENREF_12)).

**Viability**

Adult flies expressing wild type and mutant *LamC* were collected on the day of eclosion from the pupal case, designated as day zero. Approximately 30 flies were placed in each vial and transferred to a new vial every three to four days. The numbers of surviving adults were counted every two days. The numbers of surviving adults were compared to the original number of adults collected on day zero and the percentage for each day was graphed ([Melkani et al., 2013](#_ENREF_12)).

**Cytological Studies**

**Immunohistochemistry of adult hearts**: Dissected hearts from one-, three-, and five-week old adults were relaxed by a one minute treatment with 0.5 M EGTA and then fixed with 4% paraformaldehyde in PBS as previously described ([Melkani et al., 2013](#_ENREF_12)). Fixed hearts were stained with anti-Drosophila Lamin C antibodies (1:100 dilution; LC.28.26; Developmental Biology Hybridoma Bank, University of Iowa) followed by goat anti- mouse Dylight 647 (Thermofisher), anti-CncC antibodies (1:50 dilution; gift from H. Deng and T. Kerppola, University of Michigan) ([Deng & Kerppola, 2013](#_ENREF_2), [2014](#_ENREF_3)) and Alexa555-phalloidin (Invitrogen, Carlsbad, CA), which strains F-actin. Fixed hearts were also stained with anti-Ref(2)P antibody (1:100 dilution; gift from Kim Finley) ([Nezis et al., 2008](#_ENREF_13)) followed by anti-rabbit Alexa 488 (Thermofisher) and Alexa555-phalloidin (Invitrogen, Carlsbad, CA). Confocal images were taken from Zeiss LSM710 microscope (SDSU). Quantitation of aggregates in the confocal images from three to six independent adults was performed using Image J as previously reported ([Melkani et al., 2013](#_ENREF_12)).

**Lipid staining of adult abdomens:** Adults (1- and 3-weeks old) were dissected as previously described ([Melkani et al., 2013](#_ENREF_12)). After dissection, the hearts were fixed in a 4% formaldehyde solution (in 1xPBS) for 15 min. Adult abdomens were stained with Nile Red at a concentration of 10 μg/mL for 30 min in 25°C ([Lee, Bassel-Duby, & Olson, 2014](#_ENREF_10)), then washed four times for 15 minutes each and are mounted in a welled slide with Vectashield mounting medium (Vector Labs) and imaged on a Zeiss LSM710 confocal microscope (SDSU) and (University of Iowa Center for Microscopy).

**Transmission electron microscopy analysis of adult hearts**: Semi-intact heart preparations were prepared for transmission electron microscopy using a modified protocol ([Melkani et al., 2013](#_ENREF_12)). Briefly, hearts were relaxed with 10 mM EGTA for 1 minute followed by a primary fixation protocol in 3% formaldehyde, 3% glutaraldehyde in 0.1 M cacodylate buffer, pH 7.4 and a secondary fixation in 1% OsO4, 100 mM phosphate buffer, and 10 mM MgCl2, pH 7.4. The samples were blocked stained in 2% uranyl acetate and dehydrated with an acetone series, followed by orientation and embedding in Epon-filled BEEM capsules. Polymerization was performed at 60^o^C under vacuum. Thin sections (50 nm) were cut using a Diatome diamond knife on a Leica ultramicrotome and picked up on formvar-coated grids. Slices were stained with 2% uranyl acetate for 10 min and Sato’s lead stain ([Sato, 1968](#_ENREF_14)) for 2 min. Images were obtained at 120 kV on a FEI Tecnai 12 transmission electron microscope (SDSU).

**Biochemical Analyses**

**Analysis of LamC and Ref(2)P expression:** Western analysis for LamC was carried out as previously reported ([Schulze et al., 2009](#_ENREF_15)). Proteins were detected with anti-Drosophila Lamin C antibodies (LC28.26 anti-mouse IgG; University of Iowa Hybridoma Core) used at 1:5000 dilutions and antibodies to Drosophila GAPDH (University of Iowa Hybridoma Core). Chemiluminescent detection and quantitation was performed according to published procedures ([Schulze et al., 2009](#_ENREF_15)). Western and antibody dot blots were performed using dissected hearts (20-30 hearts/genotype) as described ([Melkani, Bodmer, Ocorr, & Bernstein, 2011](#_ENREF_11); [Melkani et al., 2013](#_ENREF_12)) using antibodies that recognize Ref(2)P (1;1000 dilutions; gift from Kim Finley) ([Nezis et al., 2008](#_ENREF_13)). Antibodies that recognize histone H2B (Cell Signaling, Danvers, MA) were used to control for loading.

**Analysis of glutathione levels in adult Drosophila hearts:** Adults were aged to one, seven, and 21 days. Adults were placed dorsal side down on a petroleum jelly-coated slide and then submerged with 1xPBS. An incision was made between the thorax and abdomen and the two halves were separated. The distal portion of the abdomen was removed and two incisions were made up each side in order to remove the ventral flap and extract the heart. Fifteen adults of similar age were dissected per sample. The dissected hearts were placed into 100 μl of 5% 5-sulfosalicylic acid then ground with a pestle, spun at 15,000 rpm for two minutes and the supernatant was aspirated into a fresh Eppendorf tube. The sample was flash frozen in liquid N_2_ and stored at -80. Measurements of reduced glutathione (GSH) and oxidized glutathione disulfide (GSSG) were performed as previously described ([Anderson, 1985](#_ENREF_1); [Dialynas et al., 2015](#_ENREF_5)). Briefly, twenty hearts from control and mutant flies at various age were dissected in artificial hemolymph as previously described ([Melkani et al., 2013](#_ENREF_12)) and homogenized in 5% 5-Sulfosalicylic Acid (SSA) solution. The supernatant was used to measure GSH and GSSG levels where assay activity is expressed as nanomoles of GSH per milligram soluble protein and GSSG enzyme activity as nanomoles of GSSG per milligram soluble protein (as GSH eq).

**Triglyceride analyses**: The heads of aged adults were removed by dissection and the remaining carcass was placed into 100 μl of RIPA Buffer. Each sample (3 females and 2 males per sample) was ground with a pestle for about 30 seconds and placed on ice. The samples were sonicated on an E220 Covaris Sonicator to homogeneity. In separate Eppendorf tubes, 10 μl of each homogenate was combined with 160 μl of Free Glycerol reagent (Sigma #F6428) and 40 μl of Triglyceride Reagent (Sigma #T2449). At the same time 10μl of Glycerol Standard Solution (from 2 to 2.5 mg/ml) and 10 μl of water was added to 160 μl of Free Glycerol and 40 μl of Triglyceride Reagent for the standard curve and control samples, respectively. All samples were placed into a water bath at 37°C for 10 minutes, then centrifuged at 4400 rpm for 1 minute. The samples were gently homogenized and then 150 μl of each sample was placed into a 96-well plate and analyzed at 540nm.

**Statistical analysis**

For all quantitation except lifespan analysis, statistical significance was determined using one-way analysis of variance (ANOVA) followed by Dunnett's post-hoc test to determine significance between groups with Prism 6.0 (Graph Pad) software ([Melkani et al., 2013](#_ENREF_12)). Significant differences were assumed for p<0.05. For lifespan studies, data were analyzed using the Gehan-Breslow-Wicoxon test followed by multiple comparisons between control and experimental groups. Significance was taken at p values less than the Bonferroni-corrected threshold of p<0.0125 as previously described ([Melkani et al., 2013](#_ENREF_12)).

**Supplementary Figure Legends:**

**Fig. S1** Cardiac-specific and indirect flight muscle-specific expression of wild type and mutant LamC. (a) Representative western analysis of total protein extract from hearts of one-week-old adults expressing *Hand/+* alone and in combination with wild type and mutant LamC. The membrane was stained with antibodies to LamC (top) and GAPDH (loading control) (lower). The asterisk indicates a non-specific band regularly detected with LamC antibody in protein extracts from hearts. The bar graph shows the relative levels of LamC expression after normalization to GAPDH for three westerns using independent protein samples (bottom). The value for the *Hand/+* stock set at 100%. The level of LamC expression among the stocks was not statistically different; therefore, the cardiac phenotypes are specific to the mutant LamC and not overall increased levels of LamC. (b) Quantitation of flight indexes (a measurement of adult indirect flight muscle function) for adults with cardiac-specific expression of the *Hand-Gal4* driver alone, wild type *LamC,* and mutant *LamC*. Three independent biological sets of 50 one- and three-week old adults were assayed per genotype. No statistical difference was observed among the genotypes, demonstrating that muscle defects are confined to the cardiac tissue.

**Fig. S2** Cytological defects associated with cardiac-specific expression of mutant LamC. (a and b) Merged images of cardiac tissue from the hearts of one- and three-week-old adults (one-and three-week, respectively) expressing wild type and mutant *LamC* stained with phalloidin (green), anti-LamC antibodies (red) and DAPI (blue). Cytoplasmic LamC aggregates are indicated by arrows and myofibrillar disorganization is indicated by asterisks. (c) Relative area of aggregates per total areas surveyed in confocal images of hearts from one- and three-week-old adults. Adults expressing mutant LamC had significantly more aggregated material than age-matched control \ hearts (black asterisks). Similar to the physiological heart dysfunction (Fig. 2), the cytoplasmic aggregates increased with age for both mutants (red asterisks). Statistical significance is denoted *as*: * = p < 0.05; ** = p < 0.01; *** = p < 0.001; NS = not significant.

**Fig. S3**: Cardiac-specific expression of mutant *LamC* caused mis-localization of CncC. Merged images of hearts from three-week-old adults with cardiac-specific expression of wild type and mutant LamC stained with an antibody to CncC (white, arrows), LamC (red) and DAPI (blue). The CncC antibody showed little to no staining in hearts expressing wild type *LamC*. In contrast, increased staining in both the cytoplasm and nucleus was observed in hearts expressing mutant *LamC*, relative to the control.

**Fig. S4** Cardiac-specific expression of mutant *LamC* showed limited changes in redox status and altered adipose tissue homeostasis. (a) Measurements of the redox status in hearts from one- and three-week-old (one- and three-week, respectively) adults expressing wild type and *G489V*. Measurements of reduced glutathione (GSH, top), oxidized glutathione (GSSG, middle), and the GSH/GSSG ratio (bottom) are shown. The hearts did not show little to no change in redox status for the time points analyzed, with a potential shift towards oxidative stress in the three-week-old hearts expressing G489V. (b) Dissected abdomens containing the fat bodies from 1W- and 3W-old adults expressing wild type and mutant LamC were stained with Nile Red (top). Total triglycerides were measured in adults hearts expressing wild type and mutant LaminC. Three independent sets (5 adults each) that were either eclosed (zero) to 5-week-old were used. The values were normalized to those of newly eclosed (zero) adults, set at 1**.** For both panels a and b *, **, *** and **** indicate P-values ns=not-significant, <0.05, < 0.01, 0.001, and 0.0001, respectively.

**Fig. S5** Cardiac-specific expression of *Atg1* and *CncC* RNAi suppressed heart defects caused my mutant LamC. The effects of cardiac-specific OE of *Atg1,* *Atg1 DN*, and *CncC* RNAi on the diastolic intervals (a), systolic intervals (b), diastolic diameters (c) and systolic diameters (d) in hearts of three-week-old adults (50 to 70) expressing wild type and mutant LamC were determined. Genetic modulations in hearts expressing wild type *LamC* altered a subset of the parameters depending on the genotype. Cardiac-specific knock-down of *GFP* had no impact on cardiac parameters (a-d).

**Fig. S6** Suppression of cardiac function by *Atg1* OE and *CncC* RNAi. M-mode records of dissected hearts from three-week-old females expressing wild type *LamC* and *G489V*. The restricted heart morphology caused by *G489V* and the cardiac dysrhythmias were suppressed upon *Atg1* OE and *CncC* RNAi knock-down. In contrast, expression of *Atg1 DN* did not improve cardiac restriction and, in fact, enhanced the cardiac dysrhythmia. Simultaneous expression of *Atg1* *DN* a *CncC* RNAi did not improve the morphological defects. As a control, cardiac*-*specific expression of RNAi against *GFP* caused no change in cardiac morphology.

**Fig. S7** Cardiac-specific *Atg1* OE suppressed *R205W*-induced cardiac physiological dysfunction, cytological defects, and lengthened lifespan. (a-c) *Atg1* OE in hearts of three-week-old (3W) adults (n=35 to 44, per genotype) expressing *R205W* suppressed the period defects (a), cardiac dysrhythmia (b) and enhanced cardiac performance as represented by fractional shortening (c)*.* In contrast, cardiac-specific expression of a *CncC* RNAi (KD) in three-week-old adults expressing *R205W* did not improve cardiac parameters (a-c). A *GFP* RNAi showed no effect on these cardiac parameters (a-c). Effect of these genetic modifiers on the physiology of hearts expressing wild type *LamC* was used as a control (A-C). (d) Confocal images of the hearts stained with phalloidin (green), antibody against LamC (red) and DAPI (blue) showed LamC aggregation (arrow) and myofibrillar disorganization (*) upon cardiac-specific expression of *R205W* (top). Cardiac-specific *Atg1* OE suppressed the LamC aggregates and myofibrillar disorganization (bottom). Cardiac-specific expression of a *CncC* RNAi resulted in increased LamC aggregates and further deterioration of the organization of the actin-containing myofibrils (middle). (e) The lifespan of adults (150 male and female combined data per genotype) was determined for the genotypes studied. Cardiac-specific *Atg1* OE suppressed the *R205W*-induced shortened lifespan, whereas a *CncC* RNAi did not. Statistical significance in A-C and F is denoted *as*: * = p < 0.05; ** = p < 0.01; *** = p < 0.001; NS = not significant.

**Fig. S8** An interplay between autophagy and CncC/Keap1 signaling suppressed the altered cardiac physiology caused by expression of mutant *LamC*. Simultaneous over-expression of *Atg1* OE and RNAi knock-down of *CncC* suppressed the diastolic intervals (a), systolic intervals (b), diastolic diameters (c) and systolic diameters (d) in three-week-old flies (n=33-70) expressing *G489V*. Under similar genetic conditions, expression of *Atg1 DN* in conjunction with a *CncC* RNAi resulted in further deterioration of the diastolic intervals (a), systolic intervals (b), diastolic diameters (c) and systolic diameters (d) in hearts expressing *G489V*. Similar genetic modulations in hearts expressing *LamC* had little to no effect on cardiac physiology.

**Fig. S9** An interplay between autophagy and CncC/Keap1 restored cytoplasmic localization of CncC and suppressed the elevated triglycerides caused by mutant LamC. Confocal images of hearts from three-week-old adults expressing *G489V* in combination with genetic modifiers were stained with antibodies to CncC (white), LamC (red) and DAPI (blue). Anti-CncC antibodies revealed cytoplasmic localization of CncC (arrows) in hearts expressing wild type *LamC* (a and nuclear enrichment in hearts expressing *G489V* (b). Simultaneous over-expression of *Atg1 and a* *CncC* RNAi in the presence of *G489V* caused a restoration of cytoplasmic localization (c). Simultaneous expression of *Atg1* DN and *CncC* RNA did not cause this restoration, with CncC localized to the nucleus (d). Arrowheads in panel B and RD represent mutant *LamC* aggregates. (e) Simultaneous over-expression of *Atg1* and a *CncC* RNAi suppressed the elevated levels of total triglycerides caused by *G489V*. In contrast, simultaneous expression of *Atg1 DN* and a *CncC* RNAi had no effect on triglyceride levels.

### Table S1 Summary of genetic modifiers tested for effects on *LamG489V-*induced mutant phenotypes in three-week-old aduls

| **Genotypes (*Hand-Gal4* driven *UAS-Transgenic*)** | **Cardiac physiology** | **Myofibril organization** | **Lamin aggregates and nuclear morphology** | **CncC (Nrf2) localization** | **Ref2P (P62) levels** | **Lipid profile (Nile-Red) staining** | **Life-span** |
| --- | --- | --- | --- | --- | --- | --- | --- |
| *LamC* | Normal | Organized | Absent & normal | Cytoplasmic | Normal | Normal | Normal |
| *LamG489V* | Restricted heart | Severe disorganization | Present and large | Nuclear & cytoplasmic | Upregulated | Significantly large | Shorten |
| *LamC +GFP KD* | normal | organized | Absent & Normal | Not tested | Not tested | Not tested | Normal |
| *LamG489V +GFP KD* | Restricted heart | More  disorganization | Present and large | Not tested | Not tested | Not tested | Shorten |
| *LamC + CncC KD , CG43286* | Subtle cardiac phenotype | Subtle muscle disorganization | Normal | Not tested | No change | Not affected | Subtle  shorten |
| *LamG489V + CncC (Nrf2) KD, CG43286* | Improved | More organized | Suppressed | Not tested | Upregulated | Unchanged | Not improved |
| *LamC +Keap-1 KD (CG3962)* | Subtle cardiac phenotype | Subtle muscle disorganization | Normal | No change | No change | Not affected | Subtle  shorten |
| *LamG489V + Keap-1 KD (CG3962)* | Deteriorated | More disorganized | More aggregate and large nuclei | Nuclear & cytoplasmic | Upregulated | Unchanged | Further shorten |
| *LamC +GSTD4 (CG11512)* | Subtle cardiac phenotype | Subtle muscle disorganization | Normal | Not tested | No change | Not affected | Subtle  shorten |
| *LamG489V + GSTD4 (CG11512)* | Subtle improved | More organized | Suppressed | Not tested | Upregulated | Unchanged | Not improved |
| *LamC +GSTD9 (CG10091)* | Subtle cardiac phenotype | Subtle muscle disorganization | Normal | Not tested | Not tested | Not affected | Subtle  shorten |
| *LamG489V + GSTD9 (CG10091)* | Improved cardiac physiology | More organized | Suppressed | Not tested | Not tested | Unchanged | Not improved |
| *LamC +Glutathione peroxidase (CG12013)* | Subtle cardiac phenotype | Subtle muscle disorganization | Normal | Not tested | Not tested | Not affected | Subtle  shorten |
| *LamG489V + Glutathione peroxidase (CG12013)* | Subtle improved | More organized | Suppressed | Not tested | Not tested | Unchanged | Not improved |
| *LamC +Thioredoxin reductase (CG2151)* | Subtle cardiac phenotype | Subtle muscle disorganization | Normal | Not tested | Not tested | Not affected | Subtle  shorten |
| *LamG489V + Thioredoxin reductase (CG2151)* | Subtle improved | More organized | Suppressed | Not tested | Not tested | Unchanged | Not improved |
| *LamC +Atg-1 Overexpression* | Subtle cardiac phenotype | Subtle muscle disorganization | Normal | Cytoplasmic | unchanged | Not affected | Not affected |
| *LamG489V+ Atg-1 Overexpression (OE)* | Much improved | More organized | Suppressed | Cytoplasmic | Suppressed | Uuppressed | Much improved |
| *LamC +ATG-1 Dominant negative* | Subtle restricted heart | disorganized | normal | Cytoplasmic | Unchanged | Not affected | Reduced |
| *LamG489V+ ATG-1 Dominant negative (DN)* | Deteriorated | More disorganized | More aggregate and large nuclei | Nuclear & cytoplasmic | Unchanged | Not affected | Further shorten |
| *LamC +Atg-5 Overexpression* | Normal | Normal | Normal | Not tested | Not tested | Not affected | Not affected |
| *LamG489V+ Atg-5 Overexpression* | Subtle improved | Subtle organized | Some suppression | Not tested | Not tested | Some suppression | Some improvement |
| *LamC +Atg-8a Overexpression* | Normal | Normal | Normal | Not tested | Not tested | Not affected | Not affected |
| *LamG489V+ Atg-8a Overexpression* | Subtle improved | Subtle organized | Some suppression | Not tested | Not tested | Some suppression | Some improvement |
| *LamC +Atg-1 OE + CncC KD* | Subtle cardiac phenotype | Subtle muscle disorganization | Normal | Cytoplasmic | Unchanged | unchanged | unchanged |
| *LamG489V+ Atg-1 OE & CncC KD* | Completely improved | Organized myofibrils | No aggregates and normal nuclei | Cytoplasmic and nuclear | Suppressed | Complete suppression | Completely rescued |
| *LamC +Atg-1 DN + CncC KD* | Subtle cardiac phenotype | Subtle muscle disorganization | More aggregates | Cytoplasmic | unchanged | Unchanged | Reduced |
| *LamG489V+ Atg-1 DN & CncC KD* | Deteriorated | More disorganized | More aggregate and large nuclei | Mostly nuclear | unchanged | Unchanged | Further shorten |

### Supplementary References:

### Anderson, M. E. (1985). Determination of glutathione and glutathione disulfide in biological samples. *Methods Enzymol, 113*, 548-555.

### Deng, H., & Kerppola, T. K. (2013). Regulation of Drosophila metamorphosis by xenobiotic response regulators. *PLoS Genet, 9*(2), e1003263.

### Deng, H., & Kerppola, T. K. (2014). Visualization of the Drosophila dKeap1-CncC interaction on chromatin illumines cooperative, xenobiotic-specific gene activation. *Development, 141*(16), 3277-3288.

### Dialynas, G., Flannery, K. M., Zirbel, L. N., Nagy, P. L., Mathews, K. D., Moore, S. A., & Wallrath, L. L. (2012). LMNA variants cause cytoplasmic distribution of nuclear pore proteins in Drosophila and human muscle. *Hum Mol Genet, 21*(7), 1544-1556.

### Dialynas, G., Shrestha, O. K., Ponce, J. M., Zwerger, M., Thiemann, D. A., Young, G. H., . . . Wallrath, L. L. (2015). Myopathic lamin mutations cause reductive stress and activate the nrf2/keap-1 pathway. *PLoS Genet, 11*(5), e1005231.

### Dietzl, G., Chen, D., Schnorrer, F., Su, K. C., Barinova, Y., Fellner, M., . . . Dickson, B. J. (2007). A genome-wide transgenic RNAi library for conditional gene inactivation in Drosophila. *Nature, 448*(7150), 151-156.

### Duffy, J. B. (2002). GAL4 system in Drosophila: a fly geneticist's Swiss army knife. *Genesis, 34*(1-2), 1-15.

### Fink, M., Callol-Massot, C., Chu, A., Ruiz-Lozano, P., Izpisua Belmonte, J. C., Giles, W., . . . Ocorr, K. (2009). A new method for detection and quantification of heartbeat parameters in Drosophila, zebrafish, and embryonic mouse hearts. *Biotechniques, 46*(2), 101-113.

### Han, Z., & Olson, E. N. (2005). Hand is a direct target of Tinman and GATA factors during Drosophila cardiogenesis and hematopoiesis. *Development, 132*(15), 3525-3536.

### Lee, J. H., Bassel-Duby, R., & Olson, E. N. (2014). Heart- and muscle-derived signaling system dependent on MED13 and Wingless controls obesity in Drosophila. *Proc Natl Acad Sci U S A, 111*(26), 9491-9496.

### Melkani, G. C., Bodmer, R., Ocorr, K., & Bernstein, S. I. (2011). The UNC-45 chaperone is critical for establishing myosin-based myofibrillar organization and cardiac contractility in the Drosophila heart model. *PLoS One, 6*(7), e22579.

### Melkani, G. C., Trujillo, A. S., Ramos, R., Bodmer, R., Bernstein, S. I., & Ocorr, K. (2013). Huntington's disease induced cardiac amyloidosis is reversed by modulating protein folding and oxidative stress pathways in the Drosophila heart. *PLoS Genet, 9*(12), e1004024.

### Nezis, I. P., Simonsen, A., Sagona, A. P., Finley, K., Gaumer, S., Contamine, D., . . . Brech, A. (2008). Ref(2)P, the Drosophila melanogaster homologue of mammalian p62, is required for the formation of protein aggregates in adult brain. *J Cell Biol, 180*(6), 1065-1071.

### Sato, T. (1968). A modified method for lead staining of thin sections. *J Electron Microsc (Tokyo), 17*(2), 158-159.

### Schulze, S. R., Curio-Penny, B., Speese, S., Dialynas, G., Cryderman, D. E., McDonough, C. W., . . . Wallrath, L. L. (2009). A comparative study of Drosophila and human A-type lamins. *PLoS One, 4*(10), e7564.
